# Stimulus information guides the emergence of behavior-related signals in primary somatosensory cortex during learning

**DOI:** 10.1016/j.celrep.2024.114244

**Published:** 2024-05-25

**Authors:** Mariangela Panniello, Colleen J. Gillon, Roberto Maffulli, Marco Celotto, Blake A. Richards, Stefano Panzeri, Michael M. Kohl

**Affiliations:** 1 Department of Physiology, Anatomy and Genetics, University of Oxford, Oxford OX1 3PT, UK; 2 School of Psychology and Neuroscience, University of Glasgow, Glasgow G12 8QQ, UK; 3 Optical Approaches to Brain Function Laboratory, Istituto Italiano di Tecnologia, 16163 Genova, Italy; 4 Department of Biological Sciences, University of Toronto Scarborough, Toronto, ON M1C 1A4, Canada; 5 Department of Cell & Systems Biology, University of Toronto, Toronto, ON M5S 3G5, Canada; 6 Mila, Montréal, QC H2S 3H1, Canada; 7 Neural Computation Laboratory, Center for Human Technologies, Istituto Italiano di Tecnologia, 16163 Genova, Italy; 8 Institute of Neural Information Processing, Center for Molecular Neurobiology (ZMNH), University Medical Center Hamburg-Eppendorf (UKE), 20251 Hamburg, Germany; 9 Department of Pharmacy and Biotechnology, University of Bologna, 40126 Bologna, Italy; 10 School of Computer Science, McGill University, Montréal, QC H3A 2A7, Canada; 11 Department of Neurology & Neurosurgery, McGill University, Montréal, QC H3A 1A1, Canada; 12 Learning in Machines and Brains Program, Canadian Institute for Advanced Research, Toronto, ON M5G 1M1, Canada; 13 Montreal Neurological Institute, Montréal, QC H3A 2B4, Canada

**Keywords:** mouse, sensory coding, decision-making, two-photon imaging, information theory

## Abstract

Neurons in the primary cortex carry sensory- and behavior-related information, but it remains an open question how this information emerges and intersects together during learning. Current evidence points to two possible learning-related changes: sensory information increases in the primary cortex or sensory information remains stable, but its readout efficiency in association cortices increases. We investigated this question by imaging neuronal activity in mouse primary somatosensory cortex before, during, and after learning of an object localization task. We quantified sensory- and behavior-related information and estimated how much sensory information was used to instruct perceptual choices as learning progressed. We find that sensory information increases from the start of training, while choice information is mostly present in the later stages of learning. Additionally, the readout of sensory information becomes more efficient with learning as early as in the primary sensory cortex. Together, our results highlight the importance of primary cortical neurons in perceptual learning.

## Highlights

• We quantified information in vS1 neural activity throughout sensory learning
• Sensory information is present at the start of training and increases over learning
• Choice information only emerges later in learning
• Behavioral readout of sensory information becomes more efficient with learning

## Graphical abstract

**Figure:**
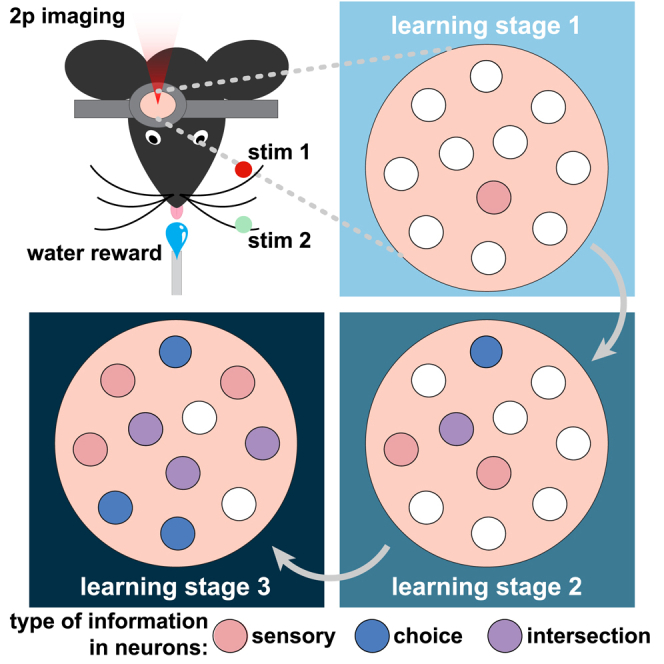

## Teaser

Panniello et al. image neuronal activity from the primary somatosensory cortex *in vivo* to study how sensory- and behavior-related information emerge and intersect with one another during sensory learning. They show that learning first improves the encoding of sensory information and then increases the efficiency of the behavioral readout of this information.

## Introduction

Neuronal activity in the vibrissal primary somatosensory cortex (vS1) of mice successfully trained on a sensory task reflects not only sensory stimuli but also various types of behavior-related information,^1^^,^^2^ including information about behavioral choice.^3^^,^^4^^,^^5^^,^^6^^,^^7^^,^^8^ Insight into learning-related changes in cortical neuronal activity is key to understanding how the brain enables flexible behavior. On an individual neuron level, a variety of learning-related changes have been observed in vS1, including sharpening of neuronal responses^3^^,^^9^ and changes in the magnitude of neuronal signals.^7^ It has been theorized that such changes serve to increase the ability of neurons to discriminate between similar pieces of information, thereby improving behavioral performance on related tasks.^10^ Yet, some studies report minimal changes in the response properties of individual vS1 neurons over the course of learning^5^^,^^11^ and instead find learning-related alterations at the population level, for example, in the relative spike timing,^12^ in neuronal gain,^3^ or in population activity correlations (for review, see Panzeri et al.^13^). The field still lacks a comprehensive picture of how stimulus- and behavior-related information emerge and are integrated with one another over time as learning takes place, and what the relative contribution of activity in individual cells vs. neuronal populations is in this process. We hypothesized that task-learning is supported by gradual changes at the individual neuron and population levels, which result in both increased information about sensory stimuli, and a more efficient use of this information to guide behavior. We anticipated that this, in turn, would contribute to generating novel, task-specific information, necessary for behavioral improvement. We tested this hypothesis by training mice on a head-fixed tactile object localization task,^14^^,^^15^ using two-photon imaging to longitudinally record the activity of excitatory neurons at different depths within layers 2 and 3 (L2/3) of vS1 before, during, and after training. For our analyses, we deployed information theory and decoding tools that are well established in neural population analyses based on electrophysiological and calcium imaging recordings.^16^^,^^17^^,^^18^^,^^19^^,^^20^ We quantified, on a trial-by-trial basis and at different stages of learning, stimulus information (MI(R;S)), behavioral choice information (MI(R;C)), and intersection information (II). II quantifies the amount of sensory information carried in the neural response that is read out to inform behavioral choice and provides insights into how information encoding may support sensory-guided behavior. We revealed that stimulus information was already present at the beginning of training, while choice information only emerged over the course of learning. Furthermore, we found that the improvement in behavioral performance was not simply accompanied by increased stimulus information but that, across learning stages, this information was more efficiently read out to instruct behavior. Finally, while changes in sensory information content were mainly shaped by changes at the individual neuron level, an increase in information encoded at the neuronal population level was more strongly associated with behavioral choice.

## Results

### Using multi-depth two-photon calcium imaging to monitor neuronal activity over the course of learning

**Figure 1:**
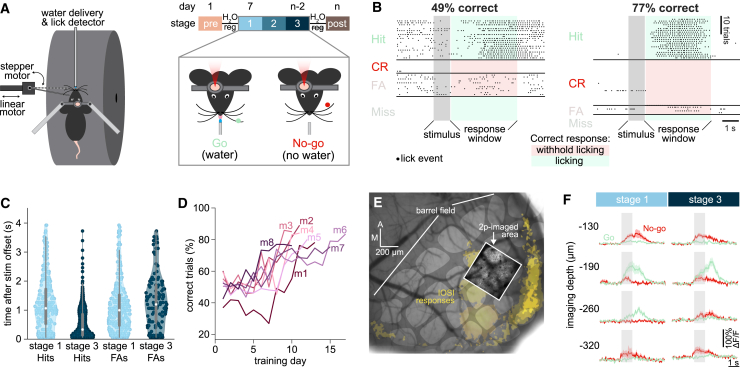
Learning of a tactile object localization task with concomitant vS1 calcium imaging (A) Experimental setup and protocol for imaging and sensory training. Mice were head-fixed but free to run on a treadmill. In each trial, a metallic pole was moved toward the left whiskers into one of two positions (anterior, Go; posterior, No-go). A spout was placed in front of the mice and used to deliver water when a lick was detected during a Go trial. Mice underwent water regulation before starting sensory training. Calcium transients in the right vS1 were recorded from the start of sensory training until the mouse achieved >70% correct responses for three consecutive days. Sensory learning was divided into three stages, based on correct performance (stage 1: pale blue, ≤55%; stage 2: blue, >55%< to ≤75%; stage 3: dark blue, >75%). (B) Lick timings during each trial of a stage 1 training session (49% correct) and a stage 3 training session (77% correct). Trials are sorted according to trial outcomes. Trials where the mouse licked only during tactile stimulation (“stimulus”) were excluded from the analysis. The dark gray shaded area indicates the time during which the pole was in contact with the whiskers. The green and red shaded areas indicate the 4-s-long response window. Correct responses included licking on Go trials (Hit) and withholding licks on No-go trials (correct rejection, CR). Incorrect responses included licking on No-go trials (false alarm, FA) and withholding licks on Go trials (Miss). (C) First lick latencies during Hit trials and FA trials for stage 1 training sessions (performance <55% correct; pale blue data points, *n* = 286 licks for Hits, 303 licks for FAs, across 8 mice) and stage 3 training sessions (performance >75% correct; dark blue data points, *n* = 949 licks for Hits, 150 licks for FAs, across 8 mice). Latency is calculated from stimulus offset. White circles indicate the median of the distribution, vertical thick gray bars indicate the 25th percentile and vertical gray lines the 75th percentile. (D) Fraction of correct responses ((Hits + CR)/total trials) in all 8 mice (m1-m8), across up to 17 days of training. (E) A representative intrinsic optical signal imaging (IOSI) image showing the location of the barrels (false-colored in light gray) corresponding to the whiskers stimulated during the procedure (yellow shading at the center of the image) in one mouse. A projection of the two-photon imaging FOV acquired throughout learning is overlaid on the IOSI image. Scale bar, 200 μm, indicating anterior (A) and medial (M) directions. (F) Mean ΔF/F_0_ across Go trials (green) and No-go trials (red) for one example neuron at each of the four cortical depths imaged (shaded areas represent SEM). In this example, the same neuron was imaged during stages 1 and 3 of learning. See also Figure S1 and Table S1.

We trained mice to learn a whisker-based object localization task^14^^,^^15^ while they were head-fixed but freely running on a cylindrical treadmill, resulting in active whisking. Mice learned to report a Go or No-go position of a vertical metal pole presented for 1–1.5 s against the left whiskers by licking for a water reward during a 4 s window starting after stimulus offset. Learning was classified into three stages, based on the percentage of correct licking responses: ≤55% (stage 1), >55 to ≤75% (stage 2), >75% (stage 3) (Figures 1A and 1B). During each of the three learning stages, we recorded the responses of excitatory neurons at four depths in the supragranular portion of the vibrissal primary somatosensory cortex (vS1), which expressed the genetically encoded calcium indicator GCaMP6s,^21^ using multi-depth two-photon calcium imaging.^22^ Learning progress was monitored using lick events. Over the course of learning, the time until the first lick after stimulus offset decreased substantially during correct Hit, but not incorrect false alarm (FA) trials (Hits: from 1.25 ± 0.06 to 0.52 ± 0.02 s; Kolmogorov-Smirnov [KS] test *p* < 0.001; FAs: from 1.13 ± 0.95 to 1.31 ± 0.08 s; KS test *p* = 0.011; mean ± SEM; Figure 1C). On average, mice took 10.4 ± 0.9 days of training to reach learning stage 3 (Figure 1D). The mean percentage of correct responses on the day of best performance was 82.7% across mice (SD: 4.06; mean d-prime: 2.31 ± 0.47). In each animal, we recorded vS1 neuronal activity in the same four fields of view (FOVs) in layers 2 and 3 (L2/3) across training sessions (Figures 1E and 1F). The overall number of neurons imaged over the course of learning stages (Table S1) as well as image quality (Figure S1) remained stable.

### Individual neurons in vS1 gain both stimulus and choice information over the course of learning

**Figure 2:**
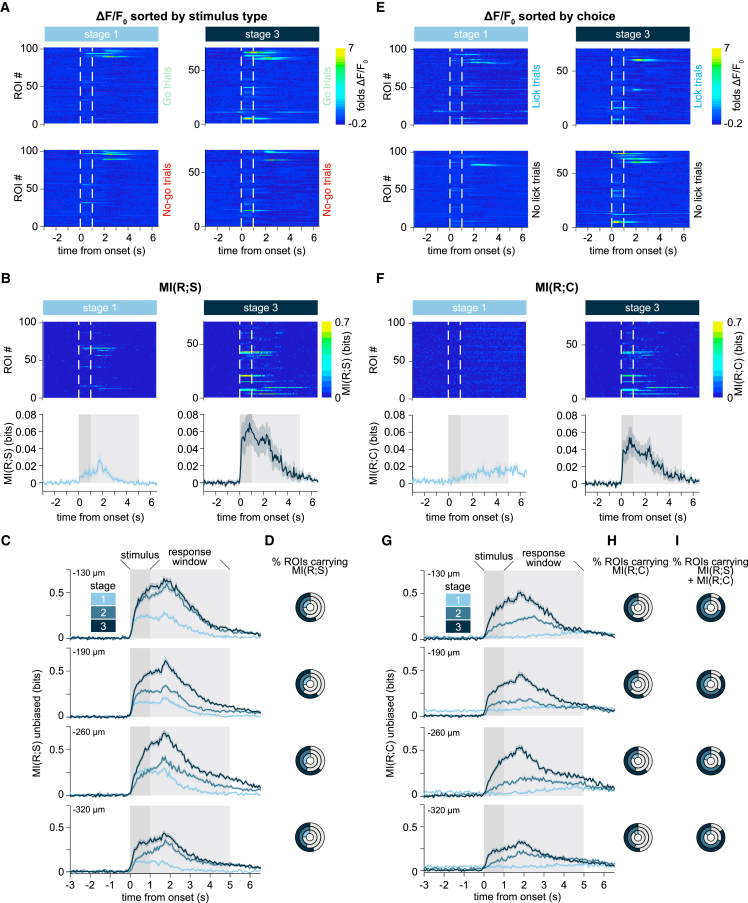
Stimulus and choice information increase over learning (A) Left: frame-by-frame mean ΔF/F_0_ across Go trials (top) and No-go trials (bottom) for all neurons (ROI no., presented in same order) in one example FOV, −190 μm below the cortical surface. Imaging was performed during learning stage 1. White lines indicate stimulus onset and offset. Right: ΔF/F_0_ activity from the same FOV, but when the mouse was in learning stage 3. (B) Left: frame-by-frame mutual information between stimulus and response (MI(R;S)) in the same neurons represented in (A) during stage 1. Right: MI(R;S) from the same FOV, when the mouse was in stage 3. Bottom left and right show the mean ± SEM MI(R;S) across all neurons in the FOV (stage 1: *n* = 102 ROIs; stage 3: *n* = 71 ROIs). The dark gray shaded area indicates stimulus duration. The pale gray shaded area indicates the (licking) response window. (C) Mean ± SEM frame-by-frame MI(R;S) across all neurons (*n* = 8 mice) at each cortical depth and for each learning stage. MI(R;S) was first averaged framewise across all neurons in the same FOV, and then averaged across all FOVs imaged at the same cortical depth and during the same learning stage (stage 1: pale blue, *n* = 461 ROIs for −130 μm, *n* = 547 for −190 μm, *n* = 525 for −260 μm, *n* = 535 for −320 μm; stage 2: blue, *n* = 709 ROIs for −130 μm, *n* = 1,073 for −190 μm, *n* = 754 for −260 μm, *n* = 621 for −320 μm; stage 3: dark blue, *n* = 395 ROIs for −130 μm, *n* = 652 for −190 μm, *n* = 461 for −260 μm, *n* = 921 for −320 μm). (D) Donut charts of the fraction of neurons carrying significant MI(R;S) at each cortical depth and at each learning stage (color coded). Full circles correspond to 100% of imaged neurons. The gray area in the charts indicates the fraction of neurons with non-significant MI(R;S) (*p* ≥ 0.05). The colored areas correspond to percent of neurons with significant MI(R;S). (E) ΔF/F_0_ activity for the same neurons as shown in (A). Here, responses were separated into Lick (top) vs. No-lick trials (bottom). (F) Same as in (B), but for mutual information between neuronal response and mouse choice (i.e., Lick vs. No-lick, MI(R;C)). (G and H) Same as in (C and D), but for MI(R;C). (I) Fraction of neurons carrying both significant MI(R;S) and significant MI(R;C), calculated over the total number of neurons carrying significant MI(R;S). See also Figures S2 and S3.

Neuronal responses to whisker touch were variable within individual FOVs, both in terms of stimulus preference (Go vs. No-go positions) and timing (early vs. late responses) at all learning stages (Figure 2A). To quantify how much information about stimulus position was carried in the activity of each imaged neuron at each time point during the trial, we calculated the frame-by-frame mutual information (MI) between a neuron’s calcium fluorescence response and the stimulus position (Go or No-go) across trials (MI(R;S); Figure 2B). We computed information for 10 s, starting 3 s before stimulus onset and ending 2 s after the end of the response window. However, quantitative analyses were run only on the first second following stimulus onset, which ended before the onset of the response window. We discretized, independently at each time point, the ΔF/F_0_ trace using two equipopulated bins, representing lower and higher neuronal activity levels (we verified that results held when discretizing with four equipopulated bins and also when operating on binarized deconvolved traces, see Figure S2). We repeated the process for each depth and learning stage. When averaging across neurons the frame-by-frame MI(R;S) values obtained for each imaged cell, we found that the overall MI(R;S) increased from stage 1 to stage 3 at all imaging depths (KS tests between stages 1 and 3, at −130, −190, −260, and −320 μm, all reported a *p* value <0.001; Figure 2C). MI(R;S) was already present during learning stage 1, as might be expected for a primary sensory region (*p* < 0.001 when compared with the null distribution, for all depths). We found that the percentage of neurons carrying significant MI(R;S) was very similar across cortical layers (−130 μm: 26.2%; −190 μm: 26.4%; −260 μm: 34.6%; −320 μm: 24.5%; Figures 2D and S3A). MI(R;S) of individual neurons was higher for superficial than deep layers, but there was 4-fold increase in MI(R;S) at −320 μm between stage 1 and stage 3 of learning (MI(R;S) stage 3/MI(R;S) stage 1 at −130 μm: 2.46, −190 μm: 3.08, −260 μm: 2.37, −320 μm: 3.88), and a doubling of the number of neurons carrying significant MI(R;S) (−130 μm: 56.9%; −190 μm: 59.1%; −260 μm: 63.1%; −320 μm: 54.6%). To test whether higher values of MI(R;S) after stimulus offset were caused by the slow GCaMP dynamics, we calculated MI(R;S) on spikes extracted from deconvolved calcium traces. This confirmed that significant sensory information is only present for approximately 1 s after stimulus onset (Figure S2).

We next asked whether individual neurons in vS1 also represent the behavioral choice to lick or withhold licking, and whether this representation changes with learning (Figure 2E). We therefore assessed MI between neural responses and choice (MI(R;C)), as above (Figures 2F and S2). Similar to MI(R;S), we found that MI(R;C) increased across learning stages. However, whereas MI(R;S) was already present in learning stage 1, the mean MI(R;C) across imaged neurons was near zero early during training, but progressively increased through the following learning stages (MI(R;C) stage 3/stage 1 at −130 μm: 10.85; −190 μm: 4.17; −260 μm: 8.84; −320 μm: 5.26; Figure 2G). This trend is reflected in the lower fraction of neurons carrying significant MI(R;C), compared with MI(R;S), in stage 1 (−130 μm: 18.5%; −190 μm: 20.1%; −260 μm: 17.3%; −320 μm: 10.5%), increasing to more than half of the imaged neurons at stage 3 (−130 μm: 56.6%; −190 μm: 56.6%; −260 μm: 61.0%; −320 μm: 51.9%; Figures 2H and S3B). As the correlation between stimulus and choice increases with learning, we subsampled trials to keep behavioral performance fixed at 75% at all learning stages. We found that MI(R;C) increased across learning stages even after this subsampling, showing that the MI(R;C) increase could not be accounted for by the stronger association between pole position and choice achieved through learning (KS tests between stage 1 and stage 3, and between stage 2 and stage 3, all reported a *p* value <0.01 at all imaged depths. KS test between stage 1 and stage 2 reported *p* > 0.05 at all depths. Figure S3C).

Moreover, by stage 3, the majority of neurons carrying significant MI(R;S) also showed significant MI(R;C) (Figure 2I), hinting at a computation taking place during learning, where primary cortical neurons encoding sensory stimulus information are recruited to inform behavioral choice as well, and contribute to task performance.

Over the course of learning, information about the tactile stimulus and behavioral choice increased as mice improved their behavioral performance. Two notable cortical layer differences could be observed: first, stimulus information stopped increasing during stage 2 in superficial L2 (−130 μm) and deep L3 (−320 μm). Second, the increase in stimulus information was strongest in deep L3 while the increase in choice information was strongest in superficial L2. Overall, these results show that, at the start of sensory training, stimulus information is already present, particularly in superficial layer 2 neurons, while choice information is absent.

### Learning-related increase in choice information is supported by population coding

Neurons in the same brain region vary in how strongly they encode sensory stimulus information, and neuronal activity in vS1 tends to be particularly sparse.^23^^,^^24^^,^^25^ Thus, we next sought to evaluate how the learning-related changes in stimulus and choice information across neurons in vS1 reflect the contribution of individual neurons to the neuronal population encoding as a whole. Calculating MI on the activity of increasing numbers of individual neurons is subject to a systematic bias due to the limited number of experimental trials available.^26^ Therefore, following established machine learning practices, we estimated the MI for groups of neurons using the MI computed on the confusion matrices obtained by training linear regression models to decode stimulus (decMI(R;S)) or choice (decMI(R;C)) from neural activity. decMI(R;S) and decMI(R;C) computed for groups of neurons offer a lower bound to the amount of stimulus and choice information encoded by the neural population.^16^ Decoders were trained with a class-balanced penalty to ensure that they learned to predict rarer classes as accurately as more frequent ones. Our decMI(R;S) and decMI(R;C) values generally correlated well with the MI(R;S) and MI(R;C) values calculated for individual neurons, indicating that the measure was reliable (Figure S4).

**Figure 3:**
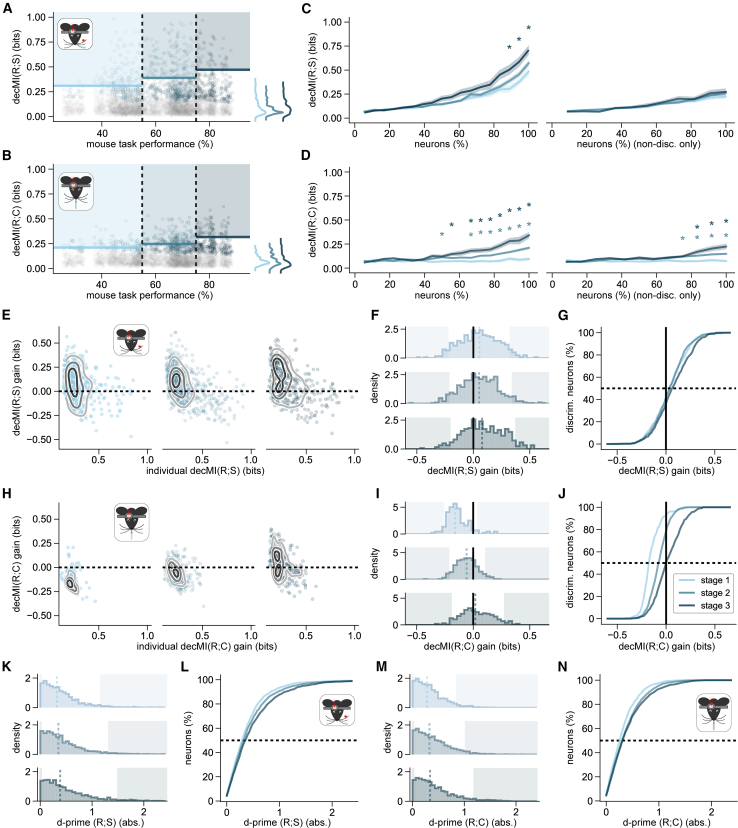
Population codes contribute more strongly to choice than to stimulus information over learning (A) decMI(R;S) (mutual information calculated on stimulus decoding confusion matrices) plotted for individual neurons (ROIs from FOVs recorded where the minimum correct/wrong task criterion was met; stage 1: pale blue, *n* = 2,512 ROIs from 42 FOVs; stage 2: blue, *n* = 3,093 ROIs from 59 FOVs; stage 3: dark blue, *n* = 1,495 ROIs from 27 FOVs), against mouse task performance (plotted with some jitter in the x dimension for visibility). The learning stages are delineated by color and vertical black dashed lines. Only discriminative neurons (decMI(R;S) *p* < 0.05, compared with the null distribution) are plotted in the learning stage color, with non-discriminative neurons plotted in gray (decMI(R;S) *p* ≥ 0.05). Colored shading indicates values above the 95th percentile (indicated by bold line). Curves on the right show the distribution across all neurons for each learning stage. (B) Same as (A), but for decMI(R;C). MI calculated on behavioral choice decoding confusion matrices (ROIs from FOVs recorded where the minimum correct/wrong task criterion and licks criteria were met; stage 1: pale blue, *n* = 1,707 ROIs from 30 FOVs; stage 2: blue, *n* = 3,030 ROIs from 59 FOVs; stage 3: dark blue, *n* = 1,495 ROIs from 27 FOVs). (C) Mean ± SEM across FOVs of population decMI(R;S) as neurons are added to the pool fed to the decoder, in order of lowest to highest individual decMI(R;S). The full pool includes either all neurons (left) or only non-discriminative neurons (right) (FOVs recorded where the minimum correct/wrong task criterion and licks criteria were met; stage 1: pale blue, 30 FOVs; stage 2: blue, 59 FOVs; stage 3: dark blue, 27 FOVs). Asterisks indicate a significant difference between stage 1 and either stage 2 or stage 3 (Mann-Whitney test *p* < 0.05 corrected, as elsewhere, for multiple comparisons). (D) Same as (C), but for decMI(R;C). (E) Discriminative neuron decMI(R;S) plotted against the gain in decMI(R;S) with respect to individual decMI(R;S), when decoders also received non-discriminative neuron responses as input. Contour lines qualitatively show data density levels. Stages 1, 2, and 3 are plotted left to right (stimulus discriminative ROIs from FOVs recorded where the minimum correct/wrong task criterion and licks criteria were met; stage 1: pale blue, *n* = 370 ROIs; stage 2: blue, *n* = 581 ROIs; stage 3: dark blue, *n* = 372 ROIs). (F) Histograms of decMI(R;S) gain (i.e., the y axis values from E), with stages 1, 2, and 3 plotted top to bottom. The solid lines mark 0 gain, whereas median gain is indicated by dashed lines, and shaded areas show values below the 5th or above the 95th percentile of the distribution. (G) Data from (F), represented as cumulative sums. The vertical solid line marks zero gain, whereas the horizontal dashed line marks the median of each distribution. As shown by the legend in (J), data for each learning stage is plotted in the stage’s color (listed in A). (H–J) Same as (E–G), but for decMI(R;C) (choice discriminative ROIs from FOVs recorded where the minimum correct/wrong task criterion and licks criteria were met; stage 1: pale blue, *n* = 135 ROIs; stage 2: blue, *n* = 355 ROIs; stage 3: dark blue, *n* = 294 ROIs). (K) Histograms of the absolute stimulus d-primes computed using ΔF/F_0_ for each neuron, with stages 1, 2, and 3 plotted top (pale blue) to bottom (dark blue) (data as in B). The median absolute d-prime is indicated by a dashed line, and shaded areas show values below the 5th or above the 95th percentile of the distribution. (L) Data from (K), represented as cumulative sums. The horizontal dashed line marks the median of each distribution. Data for each learning stage is plotted in the stage’s color (stage 1: pale blue; stage 2: blue; stage 3: dark blue). (M and N) Same as (K and L), but for absolute choice d-primes. See also Figures S4, S5, and S6.

We first sought to use decMI to evaluate the contribution of each neuron to population-level encoding of task-relevant information. We classified each neuron as “discriminative” if it carried sufficient decMI on its own to enable above-chance decoding of the trial type (i.e., above the 95th percentile of the null distribution) and “non-discriminative” otherwise.^15^ We then evaluated whether the increase in stimulus and choice information over the course of learning reflected a population level change or the emergence of a sparse set of highly informative discriminative neurons. Over the course of learning, median decMI(R;S) did not change but the percentage of discriminative neurons increased (stage 1: 16.0% ± 1.2%; stage 2: 19.5% ± 1.1%; stage 3: 24.4% ± 2.2%; mean ± SEM across depths and FOVs) and the distribution of decMI(R;S) values changed, reflecting a change in the 95th percentile (stage 1: 0.31; stage 2: 0.39; stage 3: 0.47; KS test *p* = 0.013 for stage 1 vs. stage 2, *p* = 0.005 for stage 1 vs. stage 3, but *p* > 0.05 for stage 2 vs. stage 3; Figure 3A). Similarly, for decMI(R;C) the percentage of discriminative neurons increased (stage 1: 7.4% ± 1.0%; stage 2: 11.9% ± 0.8%; stage 3: 19.6% ± 2.0%) and the distribution of decMI(R;S) values changed (95th percentile in stage 1: 0.21; stage 2: 0.25; stage 3: 0.32; KS test *p* = 0.005 for stage 1 vs. stage 2, *p* < 0.001 for stage 1 vs. stage 3, *p* = 0.019 for stage 2 vs. stage 3; Figure 3B). Together, these results indicate that the increase in stimulus and choice information observed in L2/3 of vS1 reflects both an increase in the number of discriminative neurons, as well as an increase in the information about stimulus and choice carried by the most informative neurons.

Previous work has shown that neurons, which on their own do not enable above-chance decoding of task-relevant variables, can still contribute to population encoding and improve the decoding performance of neurons that carry high information content when put together.^27^ This points to a role for non-discriminative neurons in supporting robust population codes for task-relevant information. We wanted to determine the relative importance of these non-discriminative neurons for stimulus and choice information. We therefore asked how information about stimulus and choice increased as we added neurons, from least to most informative, to the pool used for calculating decMI. For each session, we ran decoders sequentially as we added neurons with progressively increasing decMI, drawn from either the full population (Figures 3C and 3D, left) or only from the non-discriminative neuron population (Figures 3C and 3D, right). We then compared decMI(R;S) in stages 2 and 3 to stage 1 values, as neurons were added, to identify differences between stages (Mann-Whitney test, *p* < 0.05, Bonferroni corrected for all neuron % × stage comparisons). When it came to decMI(R;S), we found that, as neurons were added to the pool, decMI(R;S) tended to increase for all stages. Only once 90% or more of the entire population of recorded neurons was included, was decMI(R;S) substantially higher for stage 3 FOVs compared with stage 1 FOVs, and no differences were found for stage 2 vs. 1 FOVs (Figure 3C, left). No differences emerged between the stages when only non-discriminative neurons were included (Figure 3C, right). In contrast, during stage 1, decMI(R;C) remained low regardless of the number of neurons added to the decoder (Figure 3D). In stages 2 and 3, however, decMI(R;C) increased beyond stage 1 levels as soon as 50% to 55% of all neurons were included. In contrast to decMI(R;S), even adding only non-discriminative neurons significantly increased decMI(R;C) in stages 2 and 3 compared with stage 1 (Figure 3D). Overall, these results suggest that, as mice learn to perform the task, choice information is increasingly supported by a distributed population code. In contrast, stimulus information shows a consistent reliance on a distributed population code across learning, which is already present at the start of training.

Finally, we wanted to quantify how much stimulus or choice information individual discriminative neurons gain from the activity of a population of non-discriminative neurons. We calculated the decMI of each discriminative neuron on its own and then measured the gain in information when the decoder also received as input the neuronal activity from the FOV’s non-discriminative neurons. Including the non-discriminative population greatly increases the dimensionality of the input to the decoders, which, if the added input data is not informative, can impair a decoder’s performance. For stimulus decoding, this was the case for approximately half of all discriminative neurons, which showed a negative gain when paired with the non-discriminative population, as shown by median gains near 0 (0.05 in stage 1, 0.05 in stage 2, 0.08 in stage 3; Figures 3E–3G). The discriminative neurons that showed positive gains were generally a subset of the ones that had the lowest individual decMI(R;S) (<0.5; Figure 3E). The overall distribution of the gains in stimulus decoding showed negligible change across learning (KS test *p* = 0.006 for stage 2 vs. 3; Figures 3E–3G). In contrast, non-discriminative neurons had a much stronger effect on choice decoding by discriminative neurons. The median gain increased steadily with learning (stage 1: −0.16; stage 2: −0.06; stage 3: 0.02; Figures 3H–3J). The overall distribution of the gains in choice decoding showed a strong overall rightward shift (KS test *p* < 0.001 for all pairs of stages), with the 95th percentile increasing substantially across stages (stage 1: 0.04; stage 2: 0.11; stage 3: 0.27). Similar to stimulus decoding, the discriminative neurons that gained the most from being paired with the non-discriminative population, across all stages, were generally those with lower individual decMI(R;C) (Figure 3H).

Lastly, we sought to identify what specific single-cell or pairwise neural properties could explain changes in stimulus or choice coding with learning. We found that single-cell sensitivity, as measured by absolute d-prime values, increased across learning for stimuli (KS test stage 1 vs. 2: *p* = 0.004, stage 1 vs. 3: *p* < 0.001, stage 2 vs. 3: *p* = 0.008; Figures 3K–3L) and for choices (stage 1 vs. 2: *p* < 0.001, stage 1 vs. 3: *p* < 0.001, but stage 2 vs. 3: *p* = 0.407; Figures 3M and 3N). This was also the case when d-primes were computed on thresholded fluorescence and deconvolved spikes (Figure S5). Based on this finding, the gain in the contribution of non-discriminative neurons in choice encoding could be due to a population-wide improvement in choice sensitivity, particularly from stage 1 to 2. We also found that noise correlations improved stimulus decoding in stages 2 and 3, while decreasing choice decoding only in stage 2 (Figure S6), but only by an amount much smaller than the overall changes observed in population coding information across learning (Figure 3).

Together, these results suggest that task-relevant variables become better encoded in vS1 by the population as a whole because of a generalized increase in sensitivity of individual neurons across the population, with neurons that would not be significantly discriminative on their own contributing more effectively to the overall population code, in particular for choice encoding.

### Stimulus information increasingly guides behavioral choice throughout learning

**Figure 4:**
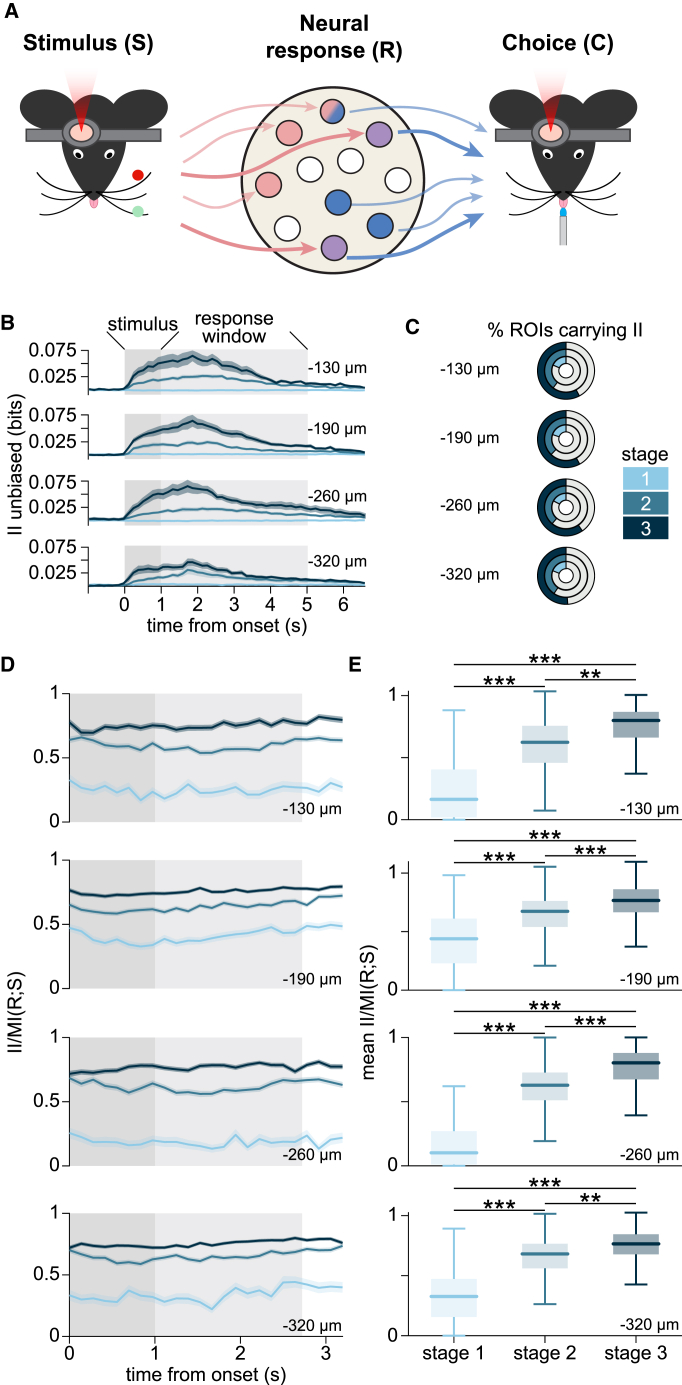
Contribution of stimulus information to behavioral choice (A) Schematic representation of the information theoretic framework showing the two stages of information processing. Stimulus encoding represents the mapping of the tactile stimuli onto the responses of neurons in L2/3 of vS1. Information readout is represented by the mapping of neuronal activity onto the mouse choice to lick or withhold licking in the presence of the tactile stimuli. Neurons, represented as circles, carry only MI(R;S) (pink) only MI(R;C) (blue), neither (white), or both. The latter neurons are represented as half blue, half pink, if they carry both MI(R;S) and MI(R;C), but not intersection information (II). However, they are represented as purple if they carry II, with thick arrows coming in from the stimulus and going out to the choice, as these neurons carry stimulus information that directly informs mouse choice. (B) Mean ± SEM frame-by-frame II across all neurons (data as in Figure 2C) at four cortical depths below pial surface (as indicated) and for each learning stage (blue curves). Dark gray shading indicates stimulus duration. Pale gray shading indicates the duration of the response window. II was first averaged framewise across all neurons in the same FOV, and then averaged across all FOVs imaged at the same cortical depth and during the same learning stage. Learning stages are color coded. (C) Donut charts of the fraction of neurons carrying significant II at each cortical depth and at each learning stage (color coded). Full circles correspond to 100% of imaged neurons. The gray area in the charts indicates the fraction of neurons with non-significant II (*p* ≥ 0.05). The colored areas correspond to percent of neurons with significant II. (D) Frame-by-frame mean II/MIRS ± SEM for all the neurons that showed significant II, at each cortical depth (data as in Figure 2C) and during each learning stage. (E) Mean II/MIRS across frames for all neurons with significant II for each learning stage and cortical depth (data as in Figure 2C). Horizontal lines indicate the median, error bars the lower and upper quartiles. Two-sided Kolmogorov-Smirnov test with Bonferroni corrections for multiple comparisons, ^∗∗^*p* < 0.01, ^∗∗∗^*p* < 0.001. See also Figures S7 and S8.

The increase in perceptual abilities when learning a sensory-guided task may be due, as traditionally hypothesized, to an increase in the sensory information encoded in early sensory cortices.^5^^,^^28^^,^^29^^,^^30^ Alternatively, it may be the consequence of an improved use of this information.^31^ To gain insights into how sensory information encoded in vS1 is used to generate accurate behavior across stages of learning, we employed II,^32^^,^^33^ an information-theoretic quantification of how much sensory information informs behavioral choices (Figure 4A). By definition, II is non-negative, on a scale of bits, and is bounded by both MI(R;S) and MI(R;C). First, we calculated the frame-by-frame II carried by each imaged neuron, across trials, at each depth and learning stage. II was, as expected, absent before stimulus onset, at all learning stages, because there was no stimulus information during this time window. After stimulus onset, II was weak during learning stage 1, but increased in stages 2 and 3 (Figure 4B). The percentage of neurons carrying significant II was low in all recorded layers in stage 1 but increased to more than half of the imaged neurons in stage 3, irrespective of cortical depth (−130 μm: 57.8%; −190 μm: 57.9%; −260 μm: 59.7%; −320 μm: 50.9%; Figures 4C and S7A). The emergence of II may be the result of two processes: (1) the increase in sensory information (MI(R;S)) encoded in neural activity over learning (Figure 2) or (2) an increase in the efficiency by which sensory information stored in vS1 is read out downstream to inform behavior. To determine the relative contribution of each process, we calculated the ratio of II/MI(R;S) for each neuron carrying significant II at each depth and learning stage. This ratio quantifies the proportion of sensory information available in neural activity that is actually read out to inform sensory behavior. We found that II/MI(R;S) increased over learning in all recorded cortical depths, peaking at ratios >0.75 in stage 3 (Figures 4D and 4E). As was done for MI(R;C), we subsampled trials to keep behavioral performance at 75% and confirmed that the increase of II across learning could not be accounted for by the stronger associations between the pole position and choice achieved through learning (Figure S7). In summary, during learning stage 1, some stimulus information is present but very little of it is directly used to inform behavioral choice. The increase in object-localization performance across learning is accompanied not only by an increase in the sensory information available in the neural activity of vS1, but also by an increase in the efficiency by which this sensory information is used to inform behavioral choices. By learning stage 3, more than 75% of the MI(R;S) could be used to guide the animal’s behavioral choice. These results were confirmed when using a simple decoder analysis^34^^,^^35^ (Figure S8).

### Task-learning produces a generalized and persistent increase in information

We have so far described the changes in information present in cortical circuits that occur when sensory stimuli are associated with a reward. To conclude, we wanted to know whether these learning-related changes in information generalize to stimuli not used in the task and persist without reward.

**Figure 5:**
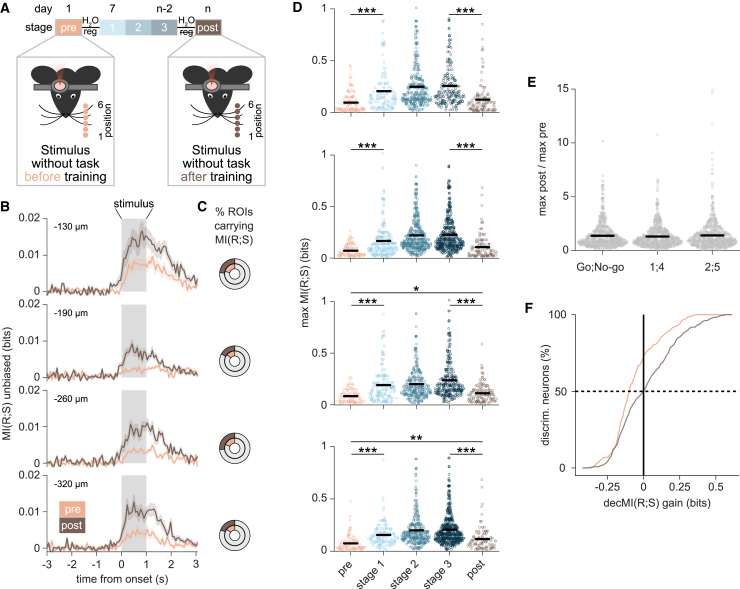
Sensory information persists in vS1 after learning (A) Schematic representation of the experimental protocol (*n* = 7 mice). ΔF/F_0_ was measured for putative neurons in vS1 while mice were presented with a metallic pole in six different positions along their anterior-posterior axis, both before (pre-training session, light orange) and after training on the pole localization task (post-training, brown). (B) Mean ± SEM frame-by-frame MI(R;S) across all neurons imaged during the pre-training and post-training sessions, for each frame and for each cortical depth (pre-training: light orange, *n* = 659 ROIs for −130 μm, *n* = 879 for −190 μm, *n* = 554 for −260 μm, *n* = 747 for −320 μm; post-training: brown, *n* = 527 ROIs for −130 μm, *n* = 765 for −190 μm, *n* = 557 for −260 μm, *n* = 567 for −320 μm). MI(R;S) was calculated on responses to stimulus positions 3 and 6 only (i.e., the pole positions used for sensory training). It was first averaged framewise across all neurons in the same FOV, and then across all FOVs imaged at the same cortical depth and during the same session. The gray shaded areas indicate stimulus duration. (C) Fraction of neurons carrying significant MI(R;S) at each cortical depth (*p* < 0.05). Full circles reflect 100% of imaged neurons. The light-gray area in each circle indicates the fraction of neurons with non-significant MI(R;S). The light orange and brown portions indicate the fraction of neurons with significant MI(R;S) during the pre-training and post-training sessions, respectively. (D) Distribution of the maximum MI(R;S) value (across frames) for each putative neuron with significant MI(R;S). Data are shown for each depth, and for each passive and active imaging session: pre-training (light orange), stage 1 training (light blue), stage 2 training (blue), stage 3 training (dark blue), and post-training (brown). Black bars indicate the mean value for each distribution. Mann-Whitney test, ^∗∗^*p* < 0.01, ^∗∗∗^*p* < 0.001. (E) Ratio between maximum MI(R;S) value during post-training and the maximum MI(R;S) value during pre-training, calculated on each putative neuron that was tracked across the two imaging sessions (*n* = 849 neurons in seven mice). MI(R;S) and ratios were calculated separately for stimuli 3 and 6 (also used during training, left), for stimuli 1 and 4 (center), and for stimuli 2 and 5 (right). Data were pooled across cortical depths. Black bars indicate the mean for each distribution. (F) Cumulative sums of gain in decMI(R;S) observed for each discriminative neuron when decoders also received non-discriminative neuron responses as input. The vertical solid line marks zero gain, the horizontal dashed line marks the median of each distribution (pre-training: light orange, *n* = 189 stimulus discriminative ROIs; post-training: brown, *n* = 235 stimulus discriminative ROIs). See also Figure S9.

In seven of the eight mice trained on the object localization task, we imaged activity in L2/3 vS1 neurons during two additional sessions (“pre-training” and “post-training”) in which sensory stimuli were presented outside of the context of the Go/No-go task, i.e., without the spout to lick or the associated water reward. The pre-training session was performed before water regulation and task training started, while the post-training session took place 2 days after the end of the task training. The stimulus was now presented in six different positions, of which positions 3 and 6 corresponded to the Go and No-go cue positions used during task training (Figure 5A). To find out how much information about stimuli 3 and 6 was present in neurons of vS1 before and after training, we calculated the frame-by-frame MI(R;S) carried by each imaged neuron, across trials, at each depth and learning stage. Average MI(R;S) across neurons was low at all cortical depths when the mice experienced the whisker stimulation for the first time (i.e., during the pre-training session). Stimulus information more than doubled after training and the fraction of neurons carrying significant MI(R;S) increased in all cortical layers (Figures 5B and 5C). The fraction of neurons carrying significant MI(R;S) during the pre-training session was consistently lower than the fraction of neurons carrying significant MI(R;S) during stage 1 of training (Figure 2D), suggesting that more neurons are recruited to encode stimulus information as soon as the stimulus-reward association is introduced.

We then asked whether sensory information improved specifically for the stimulus positions used in the object localization task, or whether the MI(R;S) increase reflected a general increase in object location information in vS1. We computed MI(R;S) on pairs of pole positions (1 and 4, 2 and 5) separated by the same distance as the Go/No-go positions 3 and 6. Before training, the percentage of significant MI(R;S) neurons was comparable across pairs of stimuli (mean across layers for 3 and 6: 17.3%; 1 and 4: 14.8%; 2 and 5: 16.9%). This percentage increased for all pairs of stimuli in the post-training session and was accompanied by a significant increase in MI(R;S) values (*p* < 0.001 for each pair of stimuli) (Figures S9A–S9D). In summary, when considering neurons carrying significant information, MI(R;S) is significantly lower pre-training than in learning stage 1 (*p* < 0.001, Mann-Whitney test), and increases across learning stages 1–3 (Figure 2), before declining post-training to levels below stage 3 (*p* < 0.001, Mann-Whitney test at all depths), but above those seen pre-training (Mann-Whitney test, *p* < 0.05 at −260 μm, *p* < 0.01 at −320 μm, ns at −130 and −190 μm) (Figure 5D). To obtain a direct measure of this MI(R;S) change for individual cells, we considered 849 neurons tracked between the pre-training and post-training imaging sessions for the three pairs of stimuli. In these neurons, MI(R;S) increased for all pairs of object locations (Kruskal-Wallis test, *p* = 0.987; Figure 5E). This post-training effect can also be seen by a stronger contribution of non-discriminative neurons to improving decMI(R;S) for individual discriminative neurons (KS test *p* < 0.001; Figure 5F and see also Figures S9E–S9H). These results demonstrate that learning-related changes in information generalize to stimuli not used in the task and persist even when the animal is no longer engaged in the task.

## Discussion

This study provides a quantitative description of stimulus and choice information and their interplay over the course of task learning in L2/3 neurons of vS1 in mice. We find that the amount of choice information encoded by the neuronal population is more strongly tied to behavioral performance than the amount of stimulus information. Furthermore, we show that choice information is increasingly supported by a population code across learning, more so than stimulus information. Finally, we present data in support of our hypothesis that the emergence of choice information in vS1 reflects a more efficient use of stimulus information, correlating with changes in behavioral performance over the course of learning. All patterns relating information values to learning and imaging depth presented in this study were fully confirmed (see Figure S2) when computing information from deconvolved calcium traces (which have the advantage of having dynamics closer to those of spiking activity compared with raw traces) rather than from ΔF/F_0_ (which has the advantage of being a less data-processed measure than deconvolved activity). The patterns were also confirmed when estimating stimulus or choice tuning with signal detection theory (d-prime) rather than with information theory. The latter analysis has the advantage of removing possible biases due to unequal choice distributions.

### Learning-related changes to stimulus and choice information in L2/3

The physiological manifestation of the perceptual changes observed in learning remains a focus of intense study. Previous reports have shown that neurons in the rodent vS1 and other primary areas not only carry sensory information but can also encode multiple task variables, from navigational signals^17^ to behavioral choice^18^^,^^19^^,^^36^^,^^37^^,^^38^^,^^39^ and expectation.^40^ Such representations may become stronger as animals learn behavioral tasks. We confirmed that both stimulus and choice information build up progressively during learning, with choice information being more dependent on task engagement than stimulus information. Traditionally, sensory learning has been considered to be the result of an improvement in the representation of sensory inputs in primary cortex. On the other hand, recent studies have found that perceptual improvements over the course of learning may correspond to an increasingly efficient readout of sensory information in higher cortical regions while sensory representations remain stable in primary areas.^31^ In our study, comparisons between the levels of MI and II over the course of learning revealed not only that stimulus information increases but also that stimulus information is more efficiently used by neurons in primary sensory cortex during the late phase of training. In other words, stimulus and choice information do not simply increase independently of one another during task learning. Instead, the increase in readout efficacy of the stimulus information leads to the increase in choice information and, likely, in behavioral performance. The enhancements of information representation and readout with learning appeared to be primarily due to a better separation of single-cell responses to sensory stimuli or choices, rather than to information-enhancing changes in the structure of noise correlations. Our findings give support to both learning-related increases in sensory information coding in primary sensory cortex and to a better readout of this information, possibly by downstream areas further up the processing hierarchy.

In our study, we use linear decoders and information theory to directly quantify information contained in neuronal activity. Our decoder analysis shows levels of stimulus and choice decoding in vS1 that are comparable with recent reports.^5^^,^^41^^,^^42^ Most studies quantified neuronal representations of information using a number of other measures, including the magnitude and frequency of neuronal activity or classification model accuracy. This difference in approach may account for some diverging observations: (1) we find that the amount of stimulus information and the number of neurons carrying it increase steadily with task training. This aligns with some previous studies,^3^^,^^7^^,^^9^ but contrasts with other reports showing that stimulus-related neuronal representations remain unchanged with learning.^5^^,^^11^ (2) We find that a large number of neurons carry significant levels of both stimulus and choice information. This significantly expands on previous work which segregated neurons based on stimulus and choice representation.^8^^,^^18^^,^^37^ Furthermore, the level of stimulus information used to inform choice in expert mice is similar to that recently reported in primary auditory cortex.^38^

### Role of single neuron vs. population codes

The relative contribution of changes in single neurons vs. the population to successful task learning is still unknown. Learning has been shown to change single neuron response patterns in vS1^3^^,^^9^ and elsewhere,^37^^,^^43^^,^^44^ but also to influence population encoding.^45^ By combining information theory with linear classifiers, we show that choice, but not stimulus, information benefits increasingly from a population code across learning. Together with the differences in the evolution of stimulus and choice information in vS1 with learning, these findings indicate that different cellular and molecular mechanisms may support stimulus and choice encoding in primary sensory cortices. Such mechanisms may include instructive top-down signals, for example, from secondary somatosensory cortex^4^ or orbitofrontal cortex,^45^ which have been shown previously to be required for choice coding. Our finding that, during initial task learning, choice information increases most in superficial L2, whereas stimulus information increases most in L3 (as also seen in Voelcker et al.^46^), hints that these top-down signals may preferentially synapse with superficial L2/3 neurons.

### Persistence of learning-related changes outside of task conditions

Lastly, we show that, following learning, when mice are re-exposed to the same stimuli outside of the context of the task, the changes in stimulus encoding observed during learning appear to persist in vS1 in a weaker, but more generalized way. Stimulus information about the task-relevant pole positions, and also nearby pole locations, increases relative to before learning began. Furthermore, it is more dependent on a population code than it was before training. This is consistent with Margolis et al.,^47^ who found that experience-induced plasticity in vS1 increased responsiveness particularly in neurons that initially showed weak stimulus responses. Together, these findings suggest that, outside of task conditions, vS1 may rely on a strengthened population code, instead of strong individual neuron responses, to continue to efficiently encode behaviorally relevant stimuli. Since this change in encoding is also context dependent, it suggests that instructive top-down signals may shape how information is encoded in the vS1 population.

Tools from information theory provided us with deeper insights into how different types of information are encoded and integrated during learning. This approach should be of great importance in identifying promising targets for manipulation to test the causal relationship between neuronal information and behavioral performance on a related task.^33^ While a growing body of work demonstrates that the manipulation of a few dozens of cortical neurons is sufficient to modulate behavior in sensory-guided tasks,^8^^,^^48^^,^^49^^,^^50^ it remains unclear why targeting so few neurons has such an effect. Our work suggests that a common feature of such neurons could be that they carry sensory information used to inform choice, offering concrete future avenues for cracking the neural code.

### Limitations of the study

Because our results are obtained with observations of neural responses, they cannot per se prove the causal contribution of the improvements in sensory coding and readout in sensory cortex neurons to improved behavioral performance. However, they provide testable hypotheses for future experiments using *in vivo* manipulation of neural activity^51^ to investigate whether these neurons carry stimulus and choice information that is necessary and/or sufficient for behavior. Furthermore, because of the slow timescale of GCaMP dynamics our work is neither intended nor suited to making precise statements about the timing of information within a behavioral trial.

## STAR★Methods

### Key resources table

**Table:** 

REAGENT or RESOURCE	SOURCE	IDENTIFIER
**Chemicals, peptides, and recombinant proteins**		

Metacam	Boehringer Ingelheim International GmbH, Ingelheim am Rhein, Germany	Meloxicam
Vetergesic	Ceva Animal Health Ltd, Amersham, UK	Buprenorphine
Marcaine	AstraZeneca, Cambridge, UK	Bupivacaine
Lacri-Lube	Allergan, UK	PL 00426/0041
IsoFlo	Zoetis, Leatherhead, UK	Isoflurane
Optical adhesive	TechOptics Ltd., UK	Norland NOA 71
Dental cement	Prestige Dental Products Ltd., UK	Super-Bond C&B
Chlorprothixene hydrochloride	Sigma-Aldrich Co Ltd, UK	C1671-1G

**Experimental models: Organisms/strains**		

Mouse: B6; DBA-Tg(tetO-GCaMP6s)2Niell/J	The Jackson Laboratory	RRID: IMSR_JAX:024742
Mouse: B6; CBA-Tg(Camk2a-tTA)1Mmay/J	The Jackson Laboratory	RRID: IMSR_JAX:003010

**Software and algorithms**		

MATLAB	MathWorks	MATLAB2021b
Python	Python Software Foundation	Python 3.9
Suite2p	Pachitariu et al.^52^	https://github.com/MouseLand/suite2p
registers2p	Pachitariu et al.^52^	https://github.com/cortex-lab/Suite2P/tree/master/registers2p
OASIS	Friedrich et al.^53^	https://github.com/j-friedrich/OASIS/releases/tag/PLoS_Comput_Biol
Neuroscience Information Toolbox (NIT)	Maffulli et al.^54^	https://gitlab.com/rmaffulli/nit
Custom analysis code	This paper	Zenodo: https://doi.org/10.5281/zenodo.10920220

**Other**		

Model 900 small animal stereotaxic instrument and mouse adaptor and ear bars	Kopf instruments, Tujunga, CA	Model 900
Small diameter cover glass 3 mm round	Harvard Apparatus, UK	64–0720
Small diameter cover glass 4 mm round	Harvard Apparatus, UK	64–0724
RS PRO Hybrid, Permanent Magnet Stepper Motor	RS Components, UK	RS Stock No.: 535–0467
Motorized linear stage	Thorlabs, UK	DDSM100/M
DC Servo Driver	Thorlabs, UK	KBD101
Allied Vision Mako U-051B high speed camera	Stemmer Imaging, UK	AV MAKO U-051B
Kowa 16 mm lens	Stemmer Imaging, UK	KOWA LM16JC
pyControl breakout board	OEPS Electrónica e Produção, Alges, Portugal	breakout board
pyControl stepper driver	OEPS Electrónica e Produção, Alges, Portugal	stepper driver
pyControl lickometer	OEPS Electrónica e Produção, Alges, Portugal	lickometer
pyControl rotary encoder adapter	OEPS Electrónica e Produção, Alges, Portugal	rotary encoder
Broadcom HEDS-5500#A02 Rotary encoder	RS Components, UK	RS Stock No.: 796-7843P
B Braun Hypodermic Needle 18 G	Fisher Scientific, UK	Product Code: 10722784
Ethafoam	Simply Foam Products Ltd., Bilston, UK	250 mm × 250 mm x 150mm
RS PRO Self-adhesive neoprene black rubber sheet	RS Components, UK	RS Stock No.: 733–6757
Ti:Sapphire laser	Spectra Physics, USA	MaiTai BB
Acousto-Optic Deflector	Photon Lines Ltd., UK	DTSX-400-980
Equilateral prism	Thorlabs, UK	SF11
Aspheric lenses	Thorlabs, UK	C330TMD-B
Moveable Objective microscope	Sutter Instruments, USA	MOM
16× Nikon CFI LWD Plan Fluorite Objective	Thorlabs, UK	N16XLWD-PF
GaAsP photomultipliers	Hamamatsu Photonics, Japan	
Piezoelectric Bimorph Bending Actuator with Wires	Thorlabs, UK	PB4NB2W
QImaging Retiga R1	Cairn Research, UK	Retiga R1
50 mm lens	Canon, UK	Canon EF 50 mm-f/1.4 USM Lens
135 mm lens	Samyang Optics	Samyang 135 mm F2.0 Manual Focus Lens for Canon

### Resource availability

#### Lead contact

Further information and requests for resources should be directed and will be fulfilled by the lead contact, Michael M. Kohl (michael.kohl@glasgow.ac.uk).

#### Materials availability

This study did not generate new unique reagents.

#### Data and code availability

• All data reported in this paper will be shared by the lead contact upon request.
• All original code used for data preprocessing and population decoding analyses, as well as the core routines computing the information-theoretic quantities, has been deposited at Zenodo and is publicly available as of the date of publication. Custom scripts calling the information theoretic analyses routines can be obtained from the lead contact upon request. The DOI of the Zenodo repository is listed in the key resource table.
• Any additional information required to reanalyze the data reported in this paper is available from the lead contact upon request.

### Experimental model and study participant details

All animal experimental procedures were approved and conducted in accordance with the United Kingdom Animals (Scientific Procedures) Act 1986 under project license P8E8BBDAD and personal licenses from the Home Office. Mice were housed in groups in a climate-controlled vivarium (lights on 7:00 to 19:00). The holding room temperature was 23 ± 1°C and humidity was set to 40 ± 10%. The experiments were conducted during the light portion of the photoperiod. Mice had *ad libitum* access to food, but access to water was restricted from one week before the start of behavioral training until the end of the training period. All weights were kept at 85–90% of the free-drinking weight for the duration of the behavioral experiments. All mice belonged to a GCaMP6s reporter line obtained by mating the TRE-GCaMP6s line (The Jackson Laboratory strain # 024742) with the CaMKII-tTA line (The Jackson Laboratory strain # 003010). The study used eight male mice, aged 9 to 12 weeks at start of the experiment (surgery, see below).

### Method details

#### Experimental design

This study did not involve randomization or blinding. We did not estimate sample size before carrying out the study.

#### Surgery

Mice underwent surgery for headbar and chronic optical window implantation. Before surgery, mice received injections of meloxicam (5 mg/kg, Metacam, Boehringer Ingelheim International GmbH, Ingelheim am Rhein, Germany) and vetergesic (0.1 mg/kg, Ceva Animal Health Ltd, Amersham, UK). They also received a marcaine (AstraZeneca, Cambridge, UK) injection under the scalp. Eye cream was applied to the eyes (lacri-Lube, Allergan, UK). Anesthesia was induced via inhalation of 4% isoflurane (Zoetis, Leatherhead, UK) at 1 L/min. When mice were fully anesthetized, they were placed in a stereotaxic frame (Kopf instruments, Tujunga, CA). Depth of anesthesia was monitored by checking pedal withdrawal reflex and respiration rate. Body temperature was kept at 37 ± 1°C. Isoflurane rate was kept at 0.8–1.2% at 0.7 L/min during surgery. A circular incision was made into the scalp, the skull was cleaned, and the periosteum removed. A 3 mm diameter craniotomy was centered over the right vS1 following stereotaxic coordinates (3.1 mm lateral from the midline and 1.3 mm posterior from the bregma suture). The dura mater was left intact. The craniotomy was then sealed with two glass coverslips (3 mm and 4 mm diameter, Harvard Apparatus, UK) glued to one another using optical adhesive (Norland, New Jersey, USA). A stainless steel headbar was cemented onto the skull using dental cement (Super-Bond C&B, Sund Medical, Japan). After surgery, mice were allowed to recover for one to two weeks before starting handling and water regulation. Handling and gentle restraint by the experimenter were performed over three days. Mice were then habituated to be headfixed under the imaging setup, and to receive water from a spout placed in front of them. This habituation phase lasted three further days, after which behavioral training started.

#### Sensory stimulation

As used in previous studies,^14^^,^^55^ tactile stimuli consisted of a small metallic pole (a blunt 18G needle, outer diameter 1.27 mm), held vertically and contacting the majority of the left whiskers of the mouse for 1 to 1.5 s at approximately 0.3 cm from the whisker pad. We used high-speed, infrared videography (AV Mako U-051B camera and Kowa 16 mm lens) to confirm that mice were able to whisk against the pole in all presented locations with the majority of their whiskers. The pole was connected to a perpendicular plastic arm mounted onto the shaft of a stepper motor (RS PRO Hybrid 535-0467; RS Components, UK). The stepper motor was mounted onto a motorized linear stage (DDSM100/M; Thorlabs, controlled by a K-Cube Brushless DC Servo Driver [KBD101; Thorlabs]), which moved the metallic pole into the calibrated touching distance toward the whiskers or away from them. The length traveled by the linear stage was identical during Go and No-go trials. During the pre-training and post-training sessions, the pole contacted the whiskers in six positions along the antero-posterior axis of the animal, separated 2.4 mm from one another. The most anterior position is denoted as position 1 throughout the text, while the most posterior is position 6. During the behavioral training phase, positions 3 and 6 were the only two used as tactile stimuli. The stepper motor only rotated between the positions once it had traveled away from the whisker pad via the linear stage. Rotating from position 1 into position 6 took approximately 90 ms longer than rotating into position 3. The sound frequency emitted consisted primarily of energy below 1 kHz, which is outside the mouse frequency hearing range.^56^ Sound intensity of the stepper motor and linear stage was <30 dB SPL. Ambient noise inside the microscope box was below 40 dB SPL. Intensity thresholds for primary auditory cortical neurons in the mouse range between 4 and 39 db SPL.^57^ Our measurements allowed us to exclude the presence of potential auditory cues during the task.

#### Behavioral training

Hardware and software for behavioral experiments were controlled through the open-source toolbox pyControl (OEPS Electrónica e Produção, Alges, Portugal).^58^ We trained mice on a whisker-based object localization Go/No-go task.^59^ Mice were free to run on a treadmill fashioned from a 24 cm diameter ethafoam cylinder covered with 3 mm-thick neoprene (Figure 1A). A rotary encoder at the hub of the wheel was used to record running activity. All mice ran before the start of the vast majority of trials or started to run as soon as the stimulus made first contact with whiskers. Running activity always resulted in concomitant active whisking, as shown in many previous studies (for review, see^60^). Therefore, virtually all trials considered in this study are recorded under active whisking conditions. As described in the previous section, the metallic pole came into contact with the whiskers for 1 to 1.5 s in one of two possible positions along the anterior-posterior axis of the mouse. The first lick latency was calculated from stimulus offset. During Go trials, mice were rewarded with an 8 μL drop of sweetened water (10% sucrose solution) when they licked from a spout during a response/licking window starting 100 ms after the retraction of the pole and lasting 4 s (Hit trials). Water was not delivered if mice licked while the pole was still in contact with the whiskers (these trials were not included in the analysis). Licks during No-go trials were considered as False Alarms (FAs) and were punished with an extended inter-trial interval (time-out). No punishment nor time-out were presented when mice did not lick during Go trials (Misses). Daily training took place in three consecutive blocks of about 16 min duration each. Across the three blocks, mice performed on average 187 ± 48 trials per training day. Learning was classified into three stages, based on the percentage of correct responses in each block: 0–55% (stage 1), 55–75% (stage 2), 75–100% (stage 3). Training ended when a mouse’s performance averaged higher than 70% across the three blocks, for three consecutive days. Only mice that performed above 70% for three consecutive days were retained for analysis (*n* = 8, of which 7 mice were used in post-training sessions). During pre-training and post-training sessions, mice were not water-regulated but had *ad libitum* access to water in their home cage. This was to avoid potential confounds related to the animals being thirsty during these sessions.

#### Two-photon imaging

Quasi-simultaneous double-plane two photon calcium imaging was performed using the set up described in detail in.^22^ Two photon excitation light was emitted by a femtosecond Ti:Sapphire laser (MaiTai BB, Spectra Physics, USA) tuned to 900 nm. Double-plane imaging was achieved using a system including a DTSX-400-980 Acousto-Optic Deflector (AOD; Photon Lines Ltd., UK), an SF11 equilateral prism (Thorlabs, UK), and two aspheric lenses (C330TMD-B, Thorlabs, UK). The laser beam was directed to the AOD, whose acoustic frequency switched between 83.5 MHz and 91.5 MHz, generating two optical paths: one leading to the nominal focal plane, and the second encompassing the aspheric lenses for refocusing onto a second focal plane, placed 130 μm below the first one. This permitted efficient acquisition of multiple planes while keeping the behavioral experiment short. During each of the three behavioral blocks (ca. 16 min, see above), we re-focused the imaging path once to acquire imaging data from fields of view (FOVs) at four depths below the cortical surface (−130, −190, −260 and −320 μm), equivalent to approximately cortical layers 2 and 3. The same FOVs were imaged in each mouse across training sessions. The two beams were then recombined through a polarizing beamsplitter. Calcium transients were acquired using a Sutter Moveable Objective microscope (MOM, Sutter, USA) controlled by ScanImage 5.2.1 software (http://scanimage.org) with minor modifications for the AOD beam steering control. The beam was scanned through an 8 kHz resonant scanner in the x-plane and a galvanometric scanning mirror in the y-plane. The resonant scanner was used in bidirectional mode, at a resolution of 512 × 512 pixels, allowing us to acquire frames at a rate of ∼15 Hz per imaging plane. A 16X/0.80W LWD immersion objective (Nikon, UK) was used. Laser power, as measured under the microscope objective, was between 80 mW and 95 mW. Emitted photons were guided through a 525/50 filter onto GaAsP photomultipliers (Hamamatsu Photonics, Japan). Neuronal fields were 400 × 400 μm in size.

#### Intrinsic Optical Signal Imaging

Intrinsic Optical Signal Imaging (IOSI) was carried out at the end of the experimental procedure to confirm that 2p imaging was performed in vS1. General anesthesia was induced with 4% isoflurane (Zoetis, Leatherhead, UK) at 1 L/min, and was then kept at 0.6–0.8% at 0.7 L/min during imaging. An *intra muscular* injection of chlorprothixene hydrochloride (1 mg/kg) was administered to inhibit whisker movements. Mice were head-fixed and placed on a heated mat. Temperature was kept at 37 ± 1°C. One whisker from the left row B, C or D was identified and threaded through a glass capillary, which was attached to a ceramic piezoelectric stimulator (e.g., PB4NB2W Piezoelectric Bimorph Bending Actuator with Wires, Thorlabs). If the surrounding whiskers touched the external side of the capillary, they were carefully trimmed using a pair of iris scissors under a dissecting microscope. A Retiga R1 camera with a 50 mm and a 135 mm lens (Nikon) attached in tandem configuration was used for imaging.^61^ Imaging was performed through the chronic cranial window previously implanted over the right parietal lobe. The whisker stimulation protocol consisted of 1 s stimulation at 10 Hz with 20 s ITI, repeated 40 times, for a total of 400 deflections. This protocol was repeated 3–4 times per mouse on different whiskers in order to map the barrel fields in vS1. For post-hoc confirmation of the imaging location in vS1, a map of the vS1 barrels obtained through IOSI was overlaid upon images of the areas investigated with 2p imaging (see, Figure 1).

### Quantification and statistical analysis

All analyses were performed using custom-written codes in MATLAB and Python. Mutual Information and intersection information calculations were carried out using the Neuroscience Information Toolbox.^54^^,^^62^ Decoder analyses were performed in Python 3.9 with custom scripts developed using the following packages: NumPy,^63^ SciPy 1.6.2,^64^ Pandas,^65^ Matplotlib,^66^ and Scikit-learn 0.24.1.^67^ The remaining analysis was using custom scripts written in MATLAB2021b.

#### Two photon imaging analysis

Raw 2p images were imported into the Suite2p software (https://github.com/MouseLand/suite2p ^52^), which performed correction for mechanical drift along the x and y axes, image segmentation, and neuronal and neuropil trace extraction. We manually inspected all regions of interest detected by the Suite2p built-in classifier, to confirm that they corresponded to neurons rather than structures such as fragments of neuronal projections or perpendicular blood vessels. For each confirmed neuron, the signal at each time frame (F*(t)*) was calculated as the average fluorescence of all pixels inside the ROI. The time series of the neuronal calcium trace and the neuropil calcium trace were exported to MATLAB for further analysis. Baseline Fluorescence (F_*0*_) was considered to be the median of the 10^th^ to the 70^th^ percentile of the fluorescence distribution across all frames acquired. Each neuron’s fluorescent trace was then corrected for the baseline using the formula: (F(t) – F_0_)/F_0_, commonly denoted as ΔF/F_0_. Subtraction of the neuropil signal was applied to each neuron’s trace as described previously,^68^ using a contamination ratio of r = 0.7. Semi-automatic ROI registration across the pre-training and post-training imaging sessions was performed using the “registers2p” package (https://github.com/cortex-lab/Suite2P/tree/master/registers2p). Although the majority of our analysis did not require systematically tracking neurons over the course of daily imaging sessions, examples of tracked neurons are given, e.g., in Figure 1G or Figure 5E. The signal-to-noise ratio (SNR) was calculated during pre-training and post-training imaging sessions on the raw fluorescent trace for each ROI. The signal was the maximum fluorescence value of the whole trace. The noise was the standard deviation (SD) of the distribution of fluorescence values recorded during the first 3 seconds of acquisition, when sensory stimulation was not yet present.^22^

Fluorescence traces data were deconvolved using first order constrained auto-regressive OASIS.^53^ For each ROI, inferred spiking activity was obtained by binarizing the resulting deconvolved trace based on a threshold set at 0.01, as used by Runyan and colleagues.^20^

#### Mutual information analyses

The information encoded by single cell responses about stimulus (MI(R;S)) and choice (MI(R;C)) has been quantified using Shannon’s Mutual Information.^16^^,^^69^ Mutual information quantifies the single-trial discriminability of stimuli or choices from neural responses. Mutual information between stimulus and response (MI(R;S)) is defined as:

(Equation 1) $$MI\left(S;R\right)=\sum_{s\in S,r\in R}p\left(s,r\right)\log_{2}\frac{p\left(s,r\right)}{p\left(s\right)p\left(r\right)}$$

Where p(s,r) denotes the joint probability of observing, in a given trial, stimulus s and neural response r, p(s) and p(r) are the respective marginal probabilities, and the sum extends to all possible values of s and r. The probabilities in Equation 1 were computed by first discretizing calcium fluorescence signals and then counting the number of occurrences of each possible pair of stimulus-response values across all trials. For analyses of information in calcium fluorescence we discretized ΔF/F_0_ into R = 2 (for main text figures) and R = 4 (for Figure S2) equipopulated bins. For analyses on deconvolved traces (for Figure S2) following previous studies,^20^^,^^38^ we binarized responses into 0 (no spikes) and 1 (one or more spikes) with a small threshold of 0.01 to remove noise in spike estimation. A similar definition holds for the mutual information between neuronal response and choice (MI(R;C)). Values of MI were corrected for limited sampling bias using the Panzeri-Treves bias correction method.^70^ Note that stimulus and choice information values are upper bounded by the stimulus or choice entropy, respectively. MI values equal to entropy indicate perfect stimulus (for stimulus information) or choices (for choice information) discriminability from neural response. Both entropies in our data were close to 1 bit in all sessions (they would be exactly 1 bit if choices or stimuli were perfectly balanced). Assessment of significance for mutual information values was performed through a permutation test of the first second of neural activity after stimulus onset (i.e., before the opening of the time window for licking). A null distribution of MI values was built through randomly permuting the neuronal response across trials.^71^ Permutation of trials was performed consistently across all time points. Permutation abolished the association between stimulus (or choice) and neuronal response while preserving the autocorrelation in the fluorescence signal. Evaluation of the significance of the MI carried by each neuron was performed as follows. For each time point, 50 permuted values of MI were calculated. Permutation was performed without correcting for limited sampling bias, owing to the stronger statistical power of non bias-corrected measures.^72^ Significance of MI for each neuron was assessed by comparing (using the Kruskal-Wallis test) the non bias-corrected MI values, with the corresponding distribution of the permuted MI values. Values of MI reported for significant neurons are corrected for limited sampling bias. Within a trial, MI(R;C) peaked about 1 s after the pole moved away from the whiskers (Figure 2G). This peak comes later than the mean first lick latency in Hit trials observed during stage 2 and 3 of learning but is in line with the first lick latency during FA trials (Figure 1D). However, since mice do not lick during Correct Rejection (CR) and Miss trials, a behavioral paradigm different from the Go/No-go used here would be required to properly evaluate any temporal coincidence between choice and peak MI(R;C). To check whether the increase in choice information across learning stages could be explained by the increased level of association between stimuli and choices with learning, we subsampled trials to equalize the behavioral performance at 75% across all learning stages, and recomputed information over these surrogate data (Figure S3C). For each ROI, a single, independent, random subsampling of trials was performed. Information quantities were calculated on the subsampled trials in the same way as for the full data.

#### Intersection information analyses

To quantify the amount of sensory information encoded by neural activity that is used to inform behavioral choices, we used Intersection Information (II).^32^ This measure is computed, using the Partial Information Decomposition (PID),^73^ from the trivariate probabilities p(s,r,c) of observing stimulus s, choice c, and neural response r in the same trial. II quantifies the part of information in neural responses that is common to both stimulus and choice information. II is computed as the minimum between two terms with similar but slightly different interpretations. The first term is SI(C;{S;R}), the information about choice C shared between stimulus S and neural response R. This quantity is obtained by computing the maximum shared dependency between S and R conditioned on C, defined as the mutual information between stimuli and neural responses minus the mutual information between stimuli and responses conditioned on choice, with the maximum computed over the space of all distributions q(s,r,c) that preserve the marginals p(s,c) and p(r,c). The second term is SI(S;{C;R}), the information about S shared between stimulus C and R. This term is obtained in a similar way by computing the maximum shared dependency between C and R, conditioned on S. Minimizing between the two terms ensures that II satisfies key properties that would be expected from a measure with this interpretation, including that independent S and R imply a zero intersection information, and that II is upper bounded by both MI(R;S) and MI(R;C). The latter property allows us to meaningfully compute the fraction II/MI(R;S) of stimulus information that is used to inform choice. We computed II discretizing probabilities p(s,r,c), correcting for limited sampling and evaluating statistical significance as explained for MI(R;S) and MI(R;C). To control for the effect of different degrees of association between stimuli and choices across learning stages on the measured II/MI(S;R) ratio, we subsampled data to equalize behavioral performance across learning stages to 75% and recomputed II/MI(R;S) on surrogate data (Figure S7), similarly to what we did for MI(R;C).

#### Decoder analyses

Linear logistic regressions were trained using 5-fold cross-validation with an L_2_ penalty. Decoders were trained separately on each session FOV, i.e., each FOV recorded during each imaging session. All decoders received, as input, frame-concatenated neuronal activity from the first second following stimulus onset, and all trial types except ones where mice licked too soon were included (Hit, FA, CR, Miss). This ensured that the inputs to the decoders did not coincide with any licking, creating confounds. Decoders trained on individual neurons received as input the ΔF/F_0_ response of a single neuron (Figures 3A, 3B, S4, and S9E), whereas decoders trained on multiple neurons received as input the concatenated ΔF/F_0_ responses of all included neurons (Figures 3C–3J, 5F, S5, S8, S9F–S9H).

Decoders were trained to predict for each trial either stimulus position (Go/No-go) (Figures 3A, 3C, 3E–3G, 5F, S4A, S6A, S6C, S6E, S8, and S9E–S9H) or choice (Lick/No-lick) (Figures 3B, 3D, 3H–3J, S4B, S6B, S6D, and S6F). To evaluate decoder performance, we computed a mutual information score on the confusion matrices obtained from the validation fold predictions (decMI).^16^ This approach allows us to estimate information content not only for individual neurons, but also for groups of neurons. We confirmed the validity of our decMI measure by comparing decMI values obtained for individual neurons to the previously computed MI measures (Figure S4). All decoders were trained using a class-balanced penalty. This ensured that decoders learned to predict rarer classes as accurately as frequent classes, thus ensuring that the decoders were not biased by the class imbalances which were necessarily very strong for choice decoding at the late learning stage. We report results grouped across depths, at each stage of learning.

When decoder performance for individual neurons was plotted against behavioral performance, jitter was added to the behavioral performance for visualization purposes to enable values for individual neurons to be distinguished. This was done by resampling the behavioral performance value for each neuron using a normal distribution centered on the true value with a standard deviation (SD) of 1.2% (Figures 3A and 3B). Since plots showing individual neuron decoder performances were very dense, distributions over decoder performances (for discriminative and non-discriminative neurons together) were computed for each stage using a Gaussian kernel density estimation and these were plotted in the right margins. These estimated distributions taper off as the density of data points gets very low, and therefore do not necessarily extend as far up as the plotted points (Figures 3A, 3B; S9E). For all decoders, to ensure that sufficient numbers of each trial type were available for training the decoders on choice decoding, only sessions with at least six Lick and six No-lick trials were included (number of session FOVs removed: eight for stage 1, on for stage 2, zero for stage 3) (Figures 3B, 3D, 3H–3J, S4B, S6B, S6D, S6F, and S8). Furthermore, to ensure that choice decoding and stimulus decoding results could be appropriately dissociated, only sessions that included at least six correct and six wrong trials were included for all decoders trained on task training sessions (number of session FOVs removed: zero for stage 1, four for stage 2, 23 for stage 3) (Figures 3A–3J, S4, S6, and S8).

Null distributions were estimated by running, for each neuron, ten 5-fold cross-validations, each computed on data where the trial types had been randomly shuffled. By aggregating decMI values resulting from shuffled data across neurons from the same session FOV, a null distribution over decoder performance was constructed for each session FOV. Neurons with decMIs above the 95^th^ percentile of the null distribution were identified as carrying significant information and labeled as “discriminative” neurons for the session FOV. Neurons that did not meet the threshold for their session FOV were labeled as “non-discriminative” (Figures 3A, 3B; S9E).

To compare decMI and MI values for each neuron, we recomputed both maximum MI and MI significance over the same trial length used for the decoders, i.e., the first second following stimulus onset. The MI(R;S) values thus obtained were then compared to decMI(R;S) for each neuron (Figure S4A), whereas MI(R;C) values were compared to decMI(R;C) values (Figure S4B). For each stage of learning, a linear regression model was fit to the data, and the goodness-of-fit was measured using the R^2^ coefficient of determination (Figure S4).

To evaluate how decoder performance changed as neurons were added to the pool of data provided to the decoders, neurons were first ordered from the lowest to highest individual decMI(R;S) (Figures 3C; S9F) or decMI(R;C) (Figure 3D). Decoders were trained to classify stimulus or choice, respectively, as neurons were added in that order, for each FOV from either the full pool of neurons (Figures 3C and 3D, left; Figure S9F, left) or the pool of non-discriminative neurons (Figures 3C and 3D, right; Figure S9F, right). This allowed us to obtain a data series for each FOV showing decoder performance as neurons were added. However, since each FOV comprised a different total number of neurons, to take the average across all the data series, we first converted the x axis for each data series from a number of neurons to a percentage of all neurons in that FOV. We then downsampled each data series to the smallest number of points common to all FOVs curves (18 percentage steps from 0 to 100% for the full population, and 11 percentage steps from 0 to 100% for the non-discriminative pool). The mean ± SEM was calculated across all downsampled FOV data series. As the data compared in this case were not normally distributed, statistical comparisons between stage 1 and 2, as well as stage 2 and 3 were computed for each data point using the non-parametric Mann-Whitney U test. As elsewhere, *p*-values were Bonferroni corrected for multiple comparisons, calculated here as the total number of comparisons for each data panel.

To determine the gain contributed by non-discriminative neurons to classification performance for discriminative neurons, in addition to training decoders on data from individual discriminative neurons, additional decoders were trained on data from each individual discriminative neuron paired with all the non-discriminative ones from the same session FOV. These decoders received as input the concatenated responses of all included neurons for each trial. The gain contributed by the non-discriminative population was then measured by, for each discriminative neuron, subtracting the decMI of the decoder trained only on the individual discriminative neuron data from the decMI of the decoder trained on data from the same discriminative neuron and the pool of non-discriminative neurons (Figures 3E, 3H; S9G). Positive gain reflected an improvement when including non-discriminative data, whereas negative gain reflected a drop in performance. Histograms over these gains were computed by binning the data into 40 bins (Figures 3F, 3I; S9H). Cumulative distributions over the same data were binned into 80 bins (Figures 3G, 3J; 5F).

To determine whether decoders trained on stimulus classification performance carried choice information, decoders were first trained on the entire pool of neurons for each session FOV to classify stimulus position (Go vs. No-go). We then sorted trials according to whether the decoder classified stimulus position correctly (“S+”, e.g., for a trial where the stimulus was classified as Go and it was Go) or incorrectly (“S-“, e.g., for a trial where the stimulus was classified as Go but it was No-go). Finally, we calculated the gain in correct choice % across session FOVs for each stage of learning when stimulus decoding was successful (% correct choice in S+ trials - % correct choice in S- trials). One-sample t-tests were then computed between both behavioral performances in order to determine whether the performance levels observed differed significantly at any learning stage for S+ vs. S- trials (Figure S8).

Absolute d-primes were computed on the same data used for the decoders (Figures 3K–3N) as follows. Neuronal responses were integrated over the first second of each trial (the same duration used for the decoders) and split into the two trial types under consideration (Go vs. No-go for stimulus, and Lick vs. No-lick for choice). Absolute d-primes were then calculated as follows:

(Equation 2) $$\left|d^{\prime }\right|=\left|\frac{\left(\mu_{2}-\mu_{1}\right)}{\sqrt{0.5\cdot \left(\sigma_{1}^{2}+\sigma_{2}^{2}\right)}}\right|$$

Absolute d-prime values were also computed on median-thresholded ΔF/F_0_ signals. Here, before integrating over the neuronal responses, the median ΔF/F_0_ across trials was computed for each neuron. All frames ΔF/F_0_ values were set to 0, except those that were above the median. Absolute d-primes were then computed as above (Figures S5A–S5D). For the absolute d-primes computed on deconvolved spikes, spikes were extracted as described in the section on two-photon imaging analysis, and absolute d-primes were computed as above (Figures S5E–S5H).

To measure the gain in decoding performance contributed by noise correlations, decMI was first computed as above for each FOV using the full neuronal population (frame-concatenated and neuron-concatenated). Next, to estimate decoding performance in the absence of noise correlations (trial-to-trial variability), trial responses were shuffled within each trial type for each neuron separately. Thus, for example, following the shuffling step, although the input responses to each now shuffled “Go trial” only comprised neuronal responses to true Go trials (not to any No-go trials), these responses came from different trials in the FOV. As a result, a decoder trained on this shuffled data could not capitalize on any consistent trial-to-trial relationships in the real data between the responses of different neurons, i.e., noise correlations. Decoders were trained for each FOV on 1000 different noise-correlation-abolishing shuffles. The median of this distribution was retained and for each FOV, a noise correlation gain was computed by subtracting the shuffle distribution median from the true decoding performance (Figures S6A and S6B). Decoders were also trained in the same manner on the pool of discriminative neurons only (Figures S6C and S6D) or of non-discriminative neurons only (Figures S6E and S6F). In all cases, one-sample t-tests were used to establish for each stage of learning whether the noise correlation gains were significantly different from 0.

Although decoder mutual information is primarily reported in this paper, we also computed balanced accuracies for the decoders (data not shown) and found that the decoding accuracies computed for discriminative neurons at all stages were comparable to those reported in previous work on decoding tactile stimuli and choice from neural activity.^3^^,^^11^^,^^42^

#### Statistical analysis

Unless otherwise specified above, differences between the distributions over population data for pairs of learning stages were evaluated using two-sided Kolmogorov-Smirnov tests with Bonferroni corrections for multiple comparisons. Corrected *p*-values <0.05 are considered significant. Mean ± standard error of the mean (SEM) is reported unless otherwise indicated. One, two and three asterisks indicate *p* < 0.05, *p* < 0.01, and *p* < 0.001 respectively.

## Supplemental information

Document S1. Figures S1–S9 and Table S1

Document S2. Article plus supplemental information
