# Supplementary material for: Stimulus information guides the emergence of behavior-related signals in primary somatosensory cortex during learning
Source: Cell Rep. 2024 May 25;43(6):114244. doi: 10.1016/j.celrep.2024.114244 (PMC11913744; doi:10.1016/j.celrep.2024.114244)
Supplement: Document S1. Figures S1–S9 and Table S1 [file mmc1.pdf]

Cell Reports, Volume *43*

Supplemental information

Stimulus information guides the emergence of behavior-related signals in primary somatosensory cortex during learning

Mariangela Panniello, Colleen J. Gillon, Roberto Maffulli, Marco Celotto, Blake A. Richards, Stefano Panzeri, and Michael M. Kohl

Supplemental Table

	Mean number ± SD of neurons recorded [number of FOVs]		
	Learning stage		
Depth below pial surface	1	2	3
-130 μm	50 ± 17 [11]	42 ± 15 [18]	32 ± 5 [12]
-190 μm	81 ± 19 [8]	70 ± 16 [16]	58 ± 25 [13]
-260 μm	48 ± 20 [11]	41 ± 16 [17]	37 ± 18 [13]
-320 μm	61 ± 23 [9]	59 ± 23 [12]	57 ± 22 [18]

Table S1. Summary of recording sessions, related to Figure 1.
The number of imaged neurons decreased slightly between stage 1 and stage 3 at -130 μm and -190 μm: $p < 0.01$ and $p < 0.05$, respectively, Mann-Whitney test. It remained stable at -260 μm and -320 μm: $p > 0.05$ for both depths, Mann-Whitney test; see **Figure S1**). The signal-to-noise ratio (SNR, see Methods) remained stable over time at each of the depths tested ($p > 0.05$), indicating that imaging quality did not deteriorate over weeks. When tested across depths, SNR was comparable, except between -130 μm and -320 μm ($p = 0.012$, Kruskal-Wallis test corrected for multiple comparisons).

Supplementary Figures

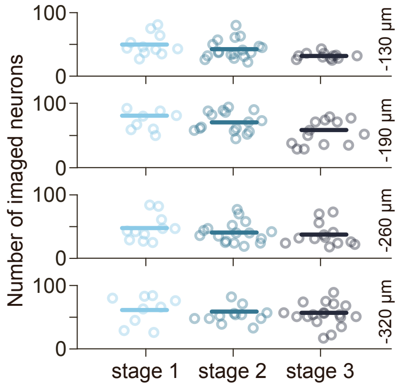

Figure S1. The number of imaged neurons remains stable across imaging sessions, related to Figure 1.

Number of neurons imaged in FOVs at each cortical depth and each learning stage (stage 1: pale blue, stage 2: blue stage 3: dark blue). Each circle represents a FOV. Horizontal lines indicate the mean across FOVs. See Table S1 for data.

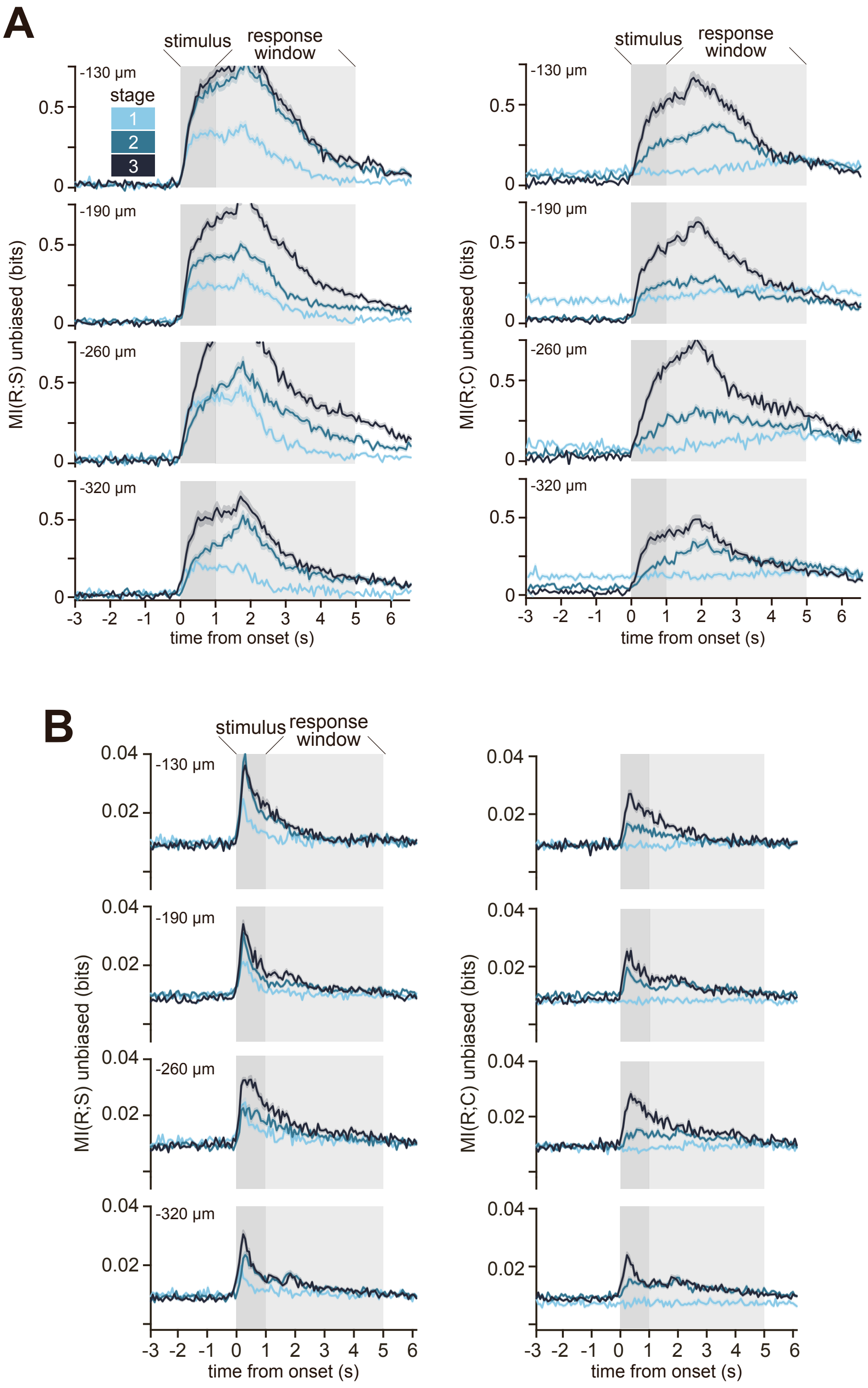

Figure S2. Stimulus and choice information increase over learning also when discretizing with four equipopulated bins and using deconvolved spikes, related to Figure 2.
(A) Mean frame-by-frame MI(R;S) and MI(R;C) across all neurons (n = 8 mice) at each cortical depth and for each learning stage as in Figure 2C and G but here $\Delta F/F_0$ was discretized at each time point into four equipopulated bins. MI(R;S) and MI(R;C) was then first averaged framewise across all neurons in the same FOV, and then averaged across all FOVs imaged at the same cortical depth and during the same learning stage.
(B) Mean frame-by-frame MI(R;S) and MI(R;C) across all neurons (n = 8 mice. Number of neurons is the same as in Figure 2C-I) at each cortical depth and for each learning stage as in Figure 2C and G, but computed on deconvolved spikes (see Methods). Colored lines indicate mean MI(R;S) or MI(R;C) across neurons, shaded areas indicate SEM (stage 1: pale blue; stage 2: blue; stage 3: dark blue).

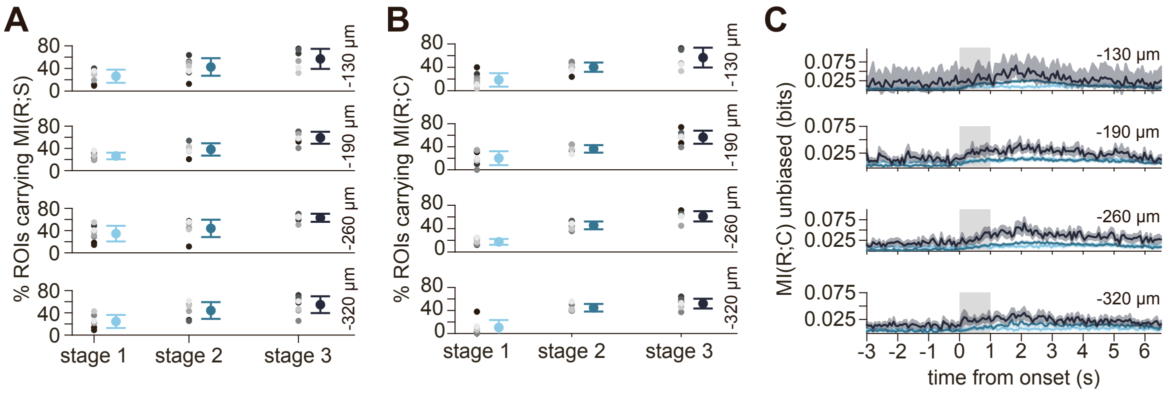

Figure S3. Further analyses on changes in information coding across learning, related to Figure 2.
(A) Gray filled circles represent the percentage of neurons carrying significant MI(R;S) in one mouse, at each learning stage and imaging depth. Dark blue, blue, and pale blue filled circles represent the mean across n = 8 mice (± SD).
(B) Same as (A), but for neurons carrying significant MI(R;C).
(C) Frame-wise MI(R;C) calculated across all imaged neurons (number of ROIs is the same as in Figure 2C-I), at each learning stage, while keeping behavioral performance fixed at 75% correct. Gray shaded areas indicate stimulus duration.

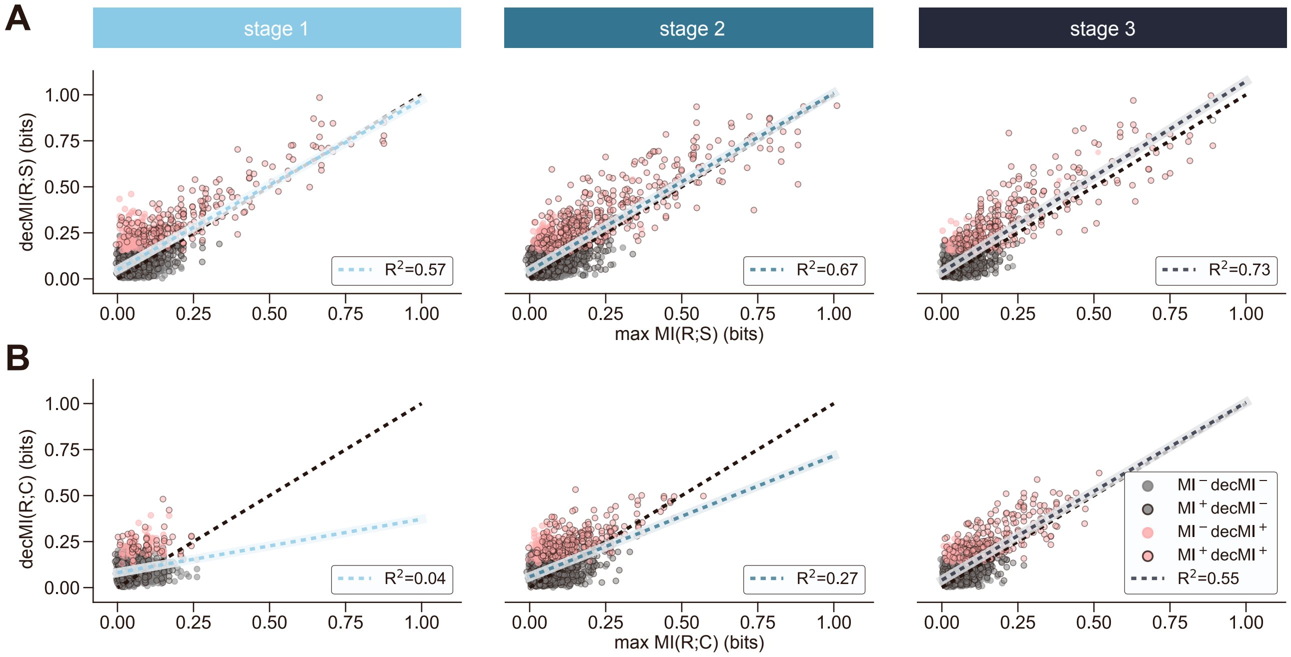

Figure S4. Mutual information calculated directly on neuronal responses correlates strongly with mutual information calculated from decoder performances for individual neurons, related to Figure 3.
(A) Maximum MI(R;S) is plotted against the MI(R;S) calculated from the decoder performances (decMI(R;S)) (both calculated over the first second following stimulus onset) for stages 1, 2 and 3 (left to right; ROIs from FOVs recorded where the minimum correct/wrong task criterion was met; stage 1: n = 2512 ROIs from 42 FOVs; stage 2: n = 3093 ROIs from 59 FOVs; stage 3: n = 1495 ROIs from 27 FOVs). As shown in the shared legend in (B), circles are plotted in shades of pink for discriminative neurons (decMI(R;S)+), and gray otherwise (decMI(R;S)-). Circles are also plotted with a dark edge if MI is significant (MI(R;S)+). The black dashed line is the identity line, and the correlation fit line is plotted in color for each stage (R^2: 0.57 in stage 1, 0.67 in stage 2, 0.73 in stage 3). The correlation between stimulus MI measures (decMI(R;S)) *vs.* max MI(R;S)) was strong for all stages, with a slope of nearly 1, pointing to a near-identity relationship. In addition, most neurons had consistent significance statuses, being significant either for both measures (MI(R;S)+decMI(R;S)+) or for neither (MI(R;S)-decMI(R;S)-) (77.5 ± 0.8% in stage 1, 71.6 ± 1.4% in stage 2, 73.2 ± 1.4% in stage 3).
(B) Same as (A), but for MI(R;C) *vs.* decMI(R;C) (ROIs from FOVs recorded where the minimum correct/wrong task criterion & licks criteria were met; stage 1: n = 1707 ROIs from 30 FOVs; stage 2: n = 3030 ROIs from 59 FOVs; stage 3: n = 1495 ROIs from 27 FOVs). The correlation fit lines are plotted in the learning stage's color (R^2: 0.04 in stage 1: pale blue, 0.27 in stage 2: blue, 0.55 in stage 3: dark blue). The correlation between decMI(R;S) and max MI(R;S)) was initially weak in stage 1, increasing substantially and reaching a near-identity relationship by stage 3. Most neurons had consistent significance statuses (80.6 ± 1.2% in stage 1, 70.1 ± 1.1% in stage 2, 73.2 ± 1.7% in stage 3).

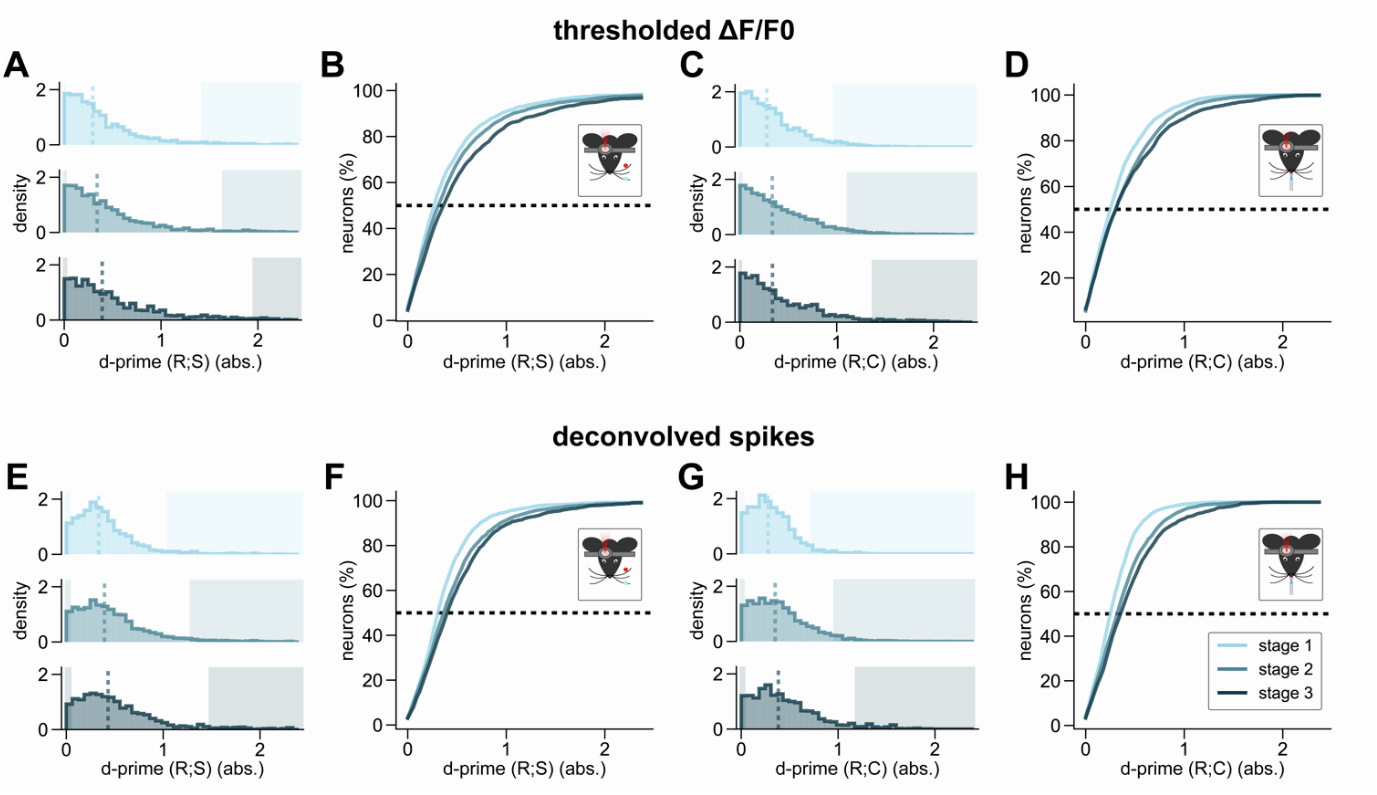

Figure S5. Sensitivity to stimulus and choice increases for individual neurons across learning, related to Figure 3.

(A) Histograms of the absolute stimulus d-primes computed for each neuron based on median-thresholded $\Delta F/F_0$, instead of $\Delta F/F_0$, with stages 1, 2 and 3 plotted top to bottom. The median across neurons of the absolute d-prime is indicated by a dashed line, and shaded areas show values below the 5th or above the 95th percentile of the distribution. KS test $p < 0.001$ for all stage comparisons, consistent with the $\Delta F/F_0$ results reported in Figure 3K-L. ROIs from FOVs recorded where the minimum correct/wrong task criterion & licks criteria were met; stage 1: n = 1707 ROIs from 30 FOVs; stage 2: n = 3030 ROIs from 59 FOVs; stage 3: n = 1495 ROIs from 27 FOVs.

(B) Data from (A), represented as cumulative sums. The horizontal black dashed line marks the median across ROIs of each distribution. Data for each learning stage is plotted in the stage's color.

(C and D) Same as (A and B), but for absolute choice d-primes. KS test stage 1 *vs.* 2: $p < 0.001$, stage 1 *vs.* 3: $p < 0.001$, but stage 2 *vs.* 3: $p = 0.083$, consistent with the $\Delta F/F_0$ results reported in Figure 3M-N.

(E and F) Same as (A and B), but absolute stimulus d-primes were calculated on deconvolved spikes, instead of $\Delta F/F_0$. KS test stage 1 *vs.* 2: $p < 0.001$, stage 1 *vs.* 3: $p < 0.001$, stage 2 *vs.* 3: $p = 0.005$, consistent with the $\Delta F/F_0$ results reported in Figure 3K-L.

(G and H) Same as (C and D), but absolute choice d-primes were calculated on deconvolved spikes, instead of $\Delta F/F_0$. KS test $p < 0.001$ for all stage comparisons, consistent with the $\Delta F/F_0$ results reported in Figure 3M-N.

Together, these results suggest that the improvements in population coding observed with learning are supported by a widespread increase of single-cell sensitivity across the population.

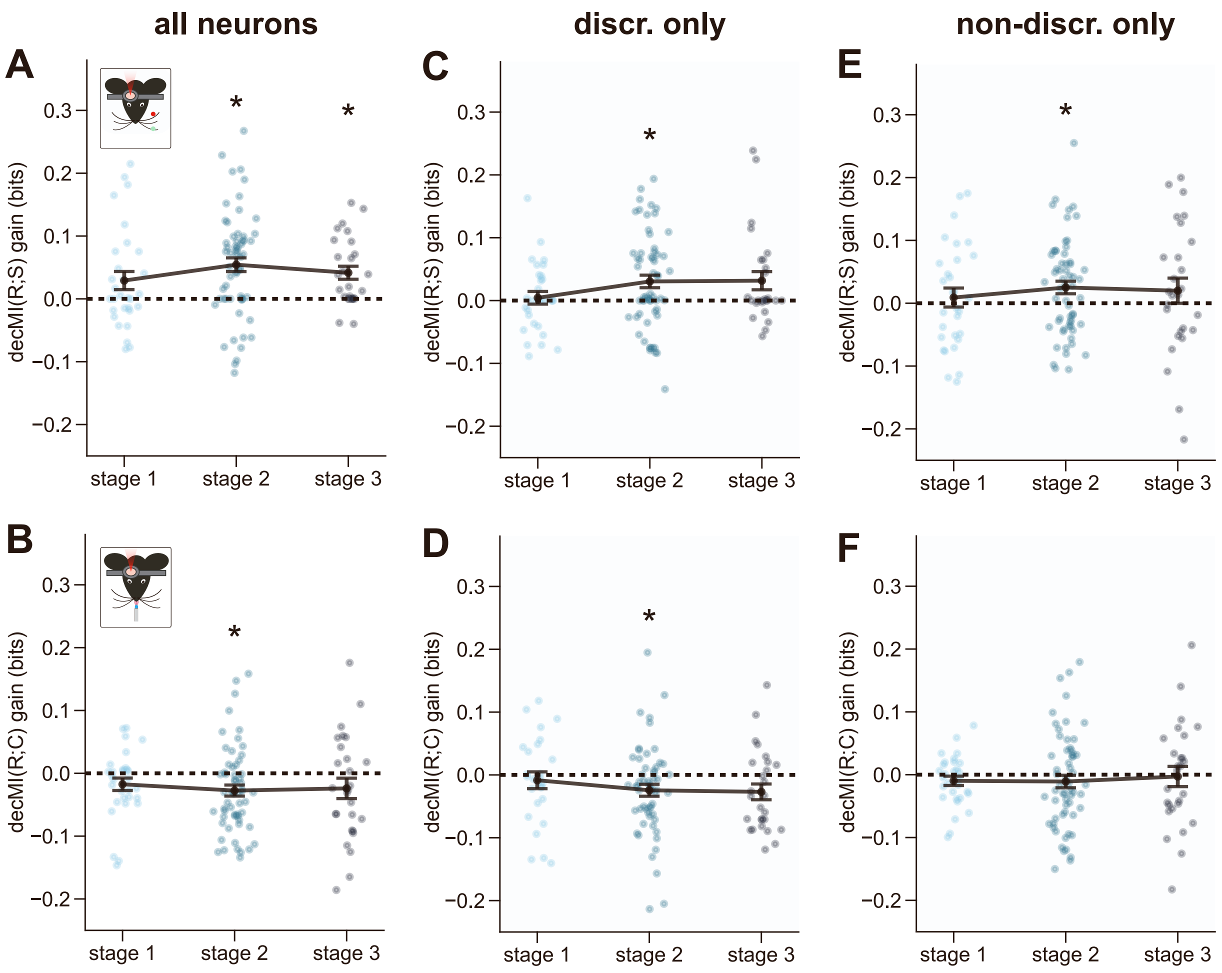

Figure S6. Contributions of noise correlations to stimulus and choice population decoding, related to Figure 3.

(A) Gain in stimulus decoding performance from noise correlations computed for each FOV (a positive gain indicates that noise correlations improve decoding, whereas a negative gain indicates that they impair decoding). Mean across FOVs is plotted, and error bars represent SEM. Circles show data for individual sessions, pooled across depths and mice (FOVs recorded where the minimum correct/wrong task criterion & licks criteria were met; stage 1: 30 FOVs; stage 2: 59 FOVs; stage 3: 27 FOVs). Asterisks indicate datapoints significantly different from 0 (one-sample t-test: $p < 0.05$). Noise correlations improved stimulus decoding in stages 2 and 3 (one-sample t-test stage 1: $p = 0.158$, stage 2: $p < 0.001$, stage 3: $p = 0.001$, with Cohen's D effect sizes 0.919 for stage 2 and 1.091 for stage 3).

(B) Same as (A), but for choice decoding. Noise correlations impaired choice decoding in stage 2 (one-sample t-test stage 1: $p = 0.263$, stage 2: $p = 0.008$, stage 3: $p = 0.454$, with Cohen's D = -0.576 in stage 2).

(C) Same as (A), but restricted to only the discriminative neurons. Data is from FOVs with stimulus discriminative neurons where the minimum correct/wrong task criterion & licks criteria were met; stage 1: 30 FOVs; stage 2: 59 FOVs; stage 3: 26 FOVs. Stage 2 gain significant as in (A), but not stage 3 gain (one-sample t-test stage 1: $p = 1.000$, stage 2: $p < 0.010$, stage 3: $p = 0.116$, with Cohen's D effect size of 0.561 for stage 2).

(D) Same as (B), but restricted to only the discriminative neurons. Data is from FOVs with choice discriminative neurons where the minimum correct/wrong task criterion & licks criteria were met; stage 1: 27 FOVs; stage 2: 57 FOVs; stage 3: 27 FOVs. Results similar to full population results in (B) (one-sample t-test stage 1: $p = 1.000$, stage 2: $p = 0.038$, stage 3: $p = 0.123$, with Cohen's D effect size of -0.482 for stage 2).

(E) Same as (A), but restricted to only the non-discriminative neurons. Data as in (C). Stage 2 gain significant as in (A and C) (one-sample t-test stage 1: $p = 1.000$, stage 2: $p = 0.045$, stage 3: $p = 0.985$, with Cohen's D effect size of 0.462 for stage 2).

(F) Same as (A), but restricted to only the non-discriminative neurons. Data as in (D). Negative gain from

noise correlations in stage 2 no longer observed (one-sample t-test stage 1: $p = 0.597$, stage 2: $p = 0.836$, stage 3: $p = 1.000$).

Overall, the changes in information due to noise correlations across learning were much smaller (on the order of 0.01 to 0.03 bits) than the overall changes observed in population information across stages of learning, particularly for choice (on the order of 0.2 bits, see Figure 3). Thus, changes in noise correlations do not appear to be a major contributor to the improvement of population codes with learning.

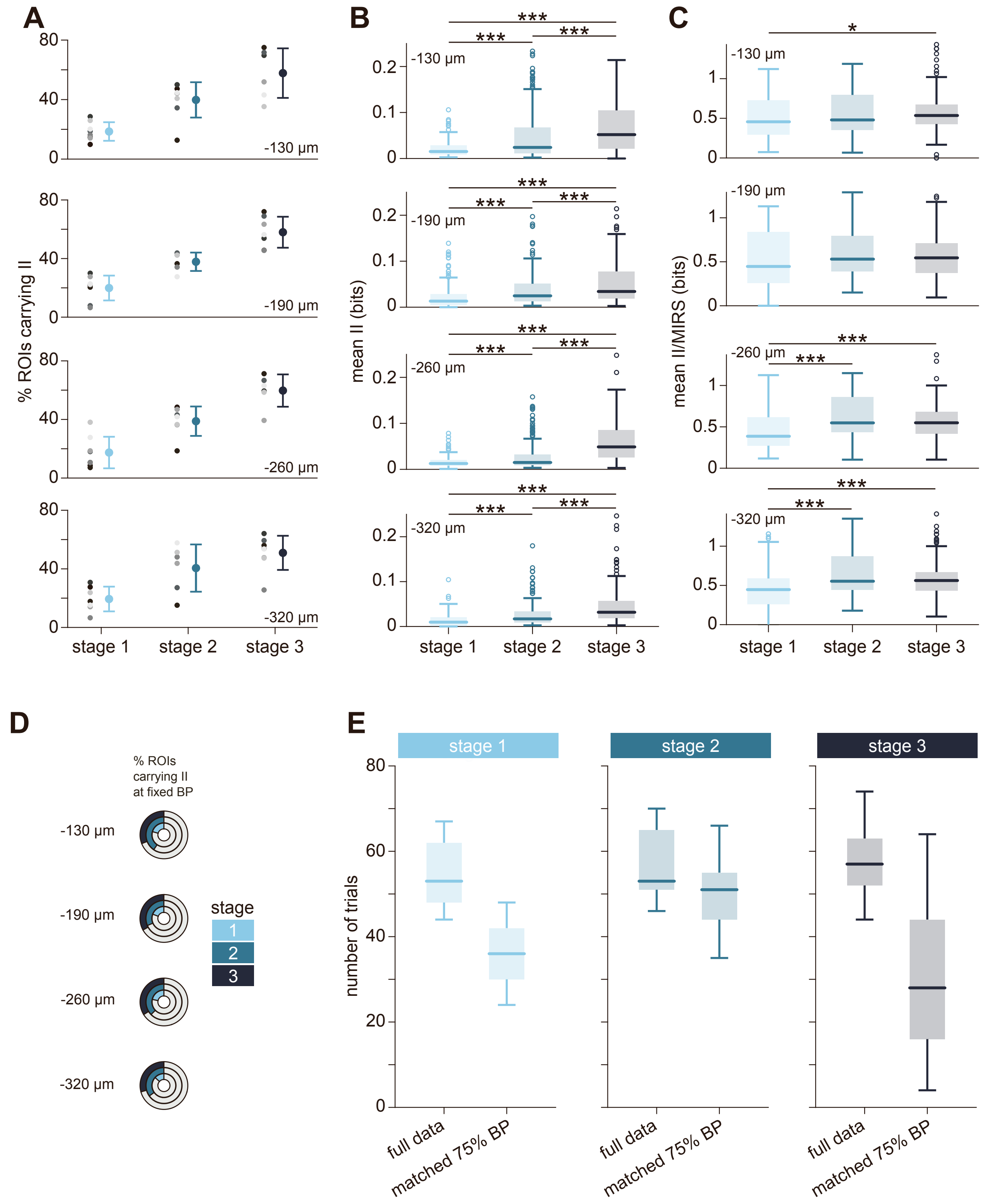

Figure S7. Increase in sensory readout efficacy in vS1 with sensory training does not depend on increased behavioral performance, related to Figure 4.

(A) Every small gray circle represents the percentage of neurons carrying significant II in one mouse, at each learning stage and imaging depth. Pale blue, blue and dark blue larger circles indicate the mean ± SD across n = 8 mice. Total number of neurons is the same as in Figure 2C-I. Proportion of ROIs carrying significant II still increases at all depths across learning when data is subsampled to control for improved behavioral performance (-130 µm: 20.6%, -190 µm: 19.2%, -260 µm: 21.5%, -320 µm: 13.4%) to stage 3 (-130 µm: 31.0%, -190 µm: 33.1%, -260 µm: 33.9%, -320 µm: 29.3%).

(B) Mean II across frames (n = 15 frames, 1 second) for all neurons with significant II for each learning stage and cortical depth when equalizing behavioral performance at 75% across all learning stages. Circles indicate outlier neurons. Horizontal lines indicate median value, and error bars indicate lower and upper quartiles. Mean II still increases at all depths across learning when data is subsampled to control for improved behavioral performance.
(C) Same as (B), but for mean II/MI(R;S). II/MI(R;S) still increases at most depths (except -190 μm) across learning when data is subsampled to control for improved behavioral performance. Two-sided KS test with Bonferroni corrections for multiple comparisons, * $p < 0.05$, *** $p < 0.001$.
(D) Donut charts of the fraction of neurons carrying significant II at each cortical depth and at each learning stage when equalizing behavioral performance at 75% across all learning stages. Full circles correspond to 100% of imaged neurons. The gray area in the charts indicates the fraction of neurons with non-significant II ($p \geq 0.05$). The colored areas correspond to % of neurons with significant II.
(E) Distributions of the number of available trials in all information-theoretical calculations (II, MI(R;S)) in all ROIs, across all depths, when considering the original behavioral response of the animal (full data) or when subsampling the trials to obtain a 75% behavioral performance (matched 75% BP).

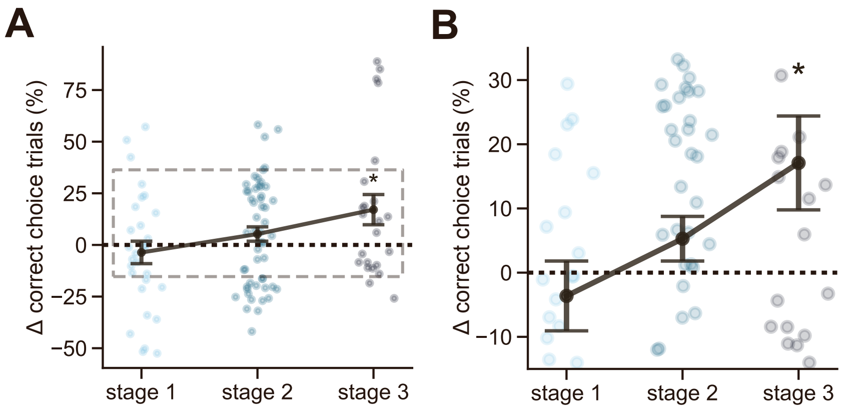

Figure S8: Decoder analysis confirms that stimulus information increasingly informs behavioral choice as learning progresses, related to Figure 4.

(A) Differences in behavioral performance (% trials in which the correct choice was made), for each FOV at each learning stage, between trials for which the stimulus was correctly versus incorrectly classified by a decoder trained on the full population. Mean across FOVs is plotted, and error bars represent SEM. Circles show data for individual sessions, pooled across depths and mice (FOVs recorded where the minimum correct/wrong task criterion & licks criteria were met; stage 1: 30 FOVs; stage 2: 59 FOVs; stage 3: 27 FOVs). Asterisks indicate datapoints significantly different from 0 (one-sample t-test: $p < 0.05$). If the sensory information encoded in neural activity was not used to inform behavioral choices, then there should be no difference in behavioral performance between correctly decoded trials (trials in which neural activity provides faithful stimulus information) and incorrectly decoded trials (where neural activity gives misleading stimulus information). In stage 1, no difference in performance was observed between correctly and incorrectly decoded trials, meaning that stimulus information that was present was not effectively used to guide choice. In stage 3, mice performed significantly better in correctly classified trials, compared to incorrectly classified ones (paired t-test $p > 0.05$ for stages 1 and 2, $p = 0.029$ with Cohen's D effect size of 0.69 for stage 3). These findings are consistent with the II results reported in Figure 4 and suggest that, as mice improved in their task performance, the sensory information encoded in vS1 neural activity was more efficiently read out by downstream structures to inform behavior.

(B) Zoomed in data from the dashed rectangle in (A).

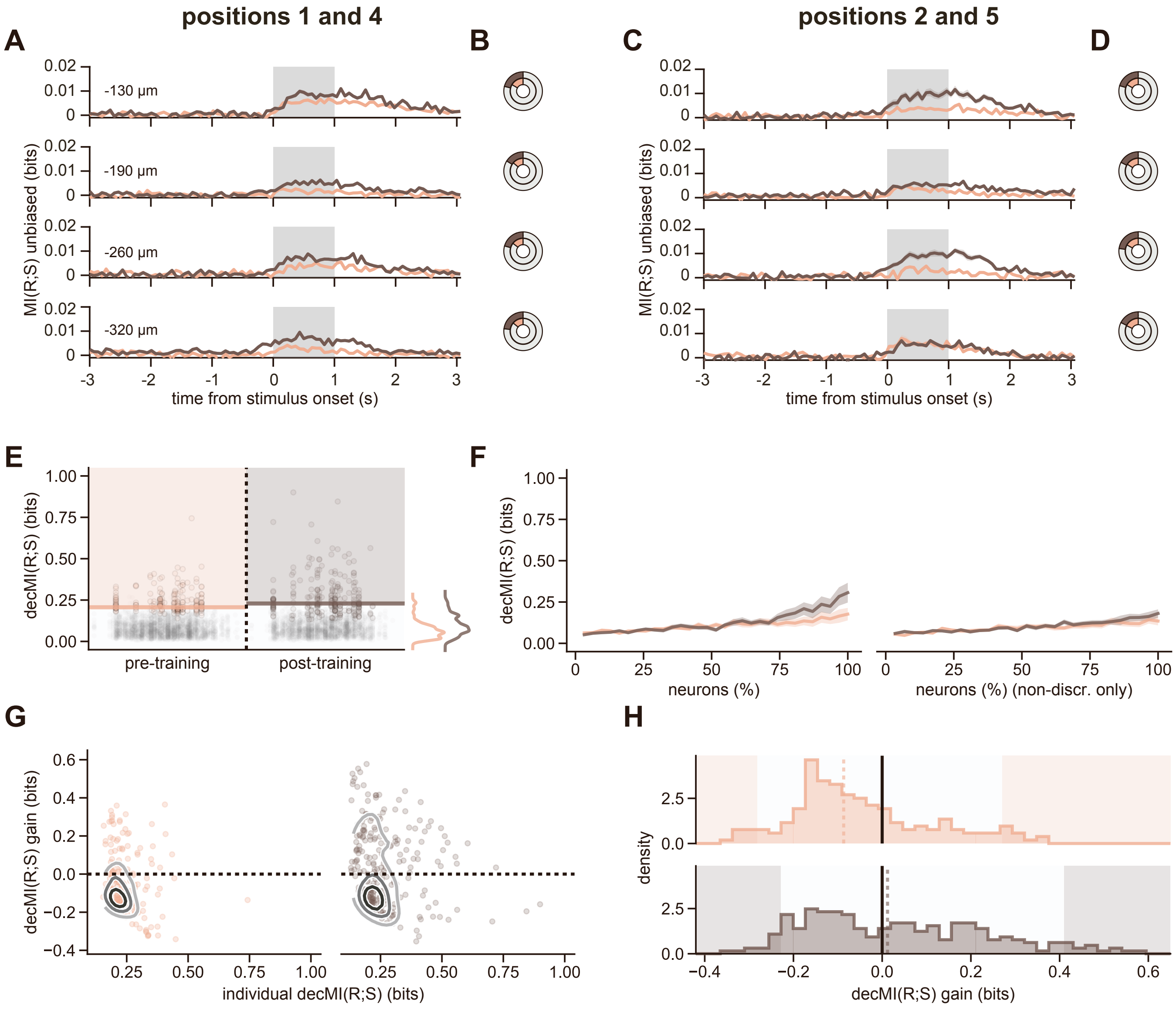

Figure S9: Sensory training improves general stimulus encoding in vS1, related to Figure 5.
(A) Mean MI(R;S) across all neurons imaged during the pre-training (light orange) and post-training (brown) session, for each frame and for each cortical depth. MI(R;S) calculated on responses to stimulus positions 1 and 4 only. The gray shaded area indicates stimulus duration.
(B) Donut charts of the fraction of neurons carrying significant MI(R;S) (stimulus positions 1 and 4) at each cortical depth ($p < 0.05$; color code as in A). Full circles correspond to 100% of imaged neurons.
(C and D) Same as (A and B), but for stimulus positions 2 and 5.
(E) decMI(R;S) (mutual information calculated on stimulus decoding confusion matrices) plotted for individual ROIs pre-training and post-training (includes all ROIs from FOVs recorded, pre-training: $n = 2839$ ROIs from 28 FOVs, post-training: $n = 2416$ ROIs from 28 FOVs; shown with jitter in the x dimension for visibility). Discriminative neurons (decMI(R;S) $p < 0.05$, compared to the chance decoding distribution) are plotted in light orange (pre-training) or brown (post-training), and non-discriminative neurons are plotted in light gray (decMI(R;S) $p \geq 0.05$). Colored shading marks values above the 95th percentile. Curves on the right show the distribution across all neurons pre- and post-training. Proportion of discriminative neurons increased modestly from 7.2 ± 1.1% pre-training to 10.4 ± 1.6% post-training, with a slight shift upward in the decMI(R;S) distribution for the population (KS test $p = 0.002$).
(F) Mean across FOVs of population decMI(R;S) as neurons are added to the pool fed to the decoder, in order of lowest to highest individual decMI(R;S). The full pool includes either all neurons (left, data as in E) or only stimulus non-discriminative neurons (right, data as in E; pre-training: light orange, post-training: brown).

Plotted lines show mean across FOVs, and shaded areas represent SEM. No datapoints showed a significant difference pre- to post-training (Mann-Whitney test, $p < 0.05$), contrasting with the increases observed during training (see Figure 3B).
(G) Discriminative neuron decMI(R;S) plotted against the gain in decMI(R;S) observed when decoders also received non-discriminative neuron responses as input. Contour lines qualitatively show data density levels. Pre-training and post-training are plotted left to right (pre-training: light orange, n = 189 ROIs; post-training: brown, n = 235 ROIs).
(H) Histograms of decMI(R;S) gain (*i.e.*, the y axis values in G), with pre-training and post-training plotted top to bottom (data as in G). Zero gain is indicated by a black line, median gain is marked as the dashed line, and shaded areas show values below the 5th or above the 95th percentile of the distribution. Gain contributed by non-discriminative neurons to stimulus information was stronger (KS test $p < 0.001$; median: -0.09 pre to 0.01 post; 5th percentile: -0.28 pre to -0.23 post; 95th percentile: 0.27 pre to 0.41 post) than during learning (see Figure 3E-G). This suggest that although the strength of stimulus information carried by individual neurons decreases when the association of the stimulus to a reward is withdrawn, the more robust encoding of stimulus information developed by the population, as a whole, is maintained.
